# GENomE wide analysis of sotalol-induced IKr inhibition during ventricular REPOLarization, “GENEREPOL study”: Lack of common variants with large effect sizes

**DOI:** 10.1371/journal.pone.0181875

**Published:** 2017-08-11

**Authors:** Joe-Elie Salem, Marine Germain, Jean-Sébastien Hulot, Pascal Voiriot, Bruno Lebourgeois, Jean Waldura, David-Alexandre Tregouet, Beny Charbit, Christian Funck-Brentano

**Affiliations:** 1 Sorbonne-Universités, UPMC Univ Paris 06, INSERM, UMRS-1166, Institute of Cardio metabolism and Nutrition (ICAN), Paris, France; 2 AP-HP, CIC-1421-Paris-Est, Pitié-Salpêtrière Hospital, Paris, France; 3 Cardiabase-Banook group, Nancy, France; Centro Cardiologico Monzino, ITALY

## Abstract

Many drugs used for non-cardiovascular and cardiovascular purposes, such as sotalol, have the side effect of prolonging cardiac repolarization, which can trigger life-threatening cardiac arrhythmias by inhibiting the potassium-channel IKr *(KCNH2)*. On the electrocardiogram (ECG), IKr inhibition induces an increase in QTc and Tpeak-Tend (TpTe) interval and a decrease of T wave maximal amplitude (TAmp). These changes vary markedly between subjects, suggesting the existence of predisposing genetic factors.

990 healthy individuals, prospectively challenged with an oral 80mg sotalol dose, were monitored for changes in ventricular repolarization on ECG between baseline and 3 hours post dosing. QTc and TpTe increased by 5.5±3.5% and 15±19.6%, respectively, and TAmp decreased by 13.2±15.5%. A principal-component analysis derived from the latter ECG changes was performed. A random subsample of 489 individuals were subjected to a genome-wide-association analysis where 8,306,856 imputed single nucleotide polymorphisms (SNPs) were tested for association with QTc, TpTe and TAmp modulations, as well their derived principal-components, to search for common genetic variants associated with sotalol-induced IKr inhibition. None of the studied SNPs reached the statistical threshold for genome-wide significance.

This study supports the lack of common variants with larger effect sizes than one would expect based on previous ECG genome-wide-association studies.

**Clinical trial registration**: ClinicalTrials.gov NCT00773201

## Introduction

Torsade de pointes (TdP) is a life-threatening arrhythmia occurring in the setting of marked prolongation of the ventricular repolarization, as assessed by prolongation of QT interval on the electrocardiogram. Long QT syndromes (LQTS) can be inherited (called « congenital long QT ») or induced following drug administration (« acquired or drug-induced long QT »). Drug-induced LQTS (diLQTS) and drug-induced Torsade de pointes (diTdP) are one of the most common causes of drug withdrawal from market or clinical development, causing major setbacks to drug discovery efforts and exposing patients to potentially dangerous drugs. The acquired LQTS is triggered by a multiplicity of drugs, either used for cardiovascular and non-cardiovascular purposes, such as anti-arrhythmic, psychiatric or anti-infective agents [1]. The main electrophysiological process leading to drug-induced long QT (diLQT) is inhibition of the transmembrane potassium channel IKr [1,2] (encoded by *KCNH2*), which results in prolongation of ventricular repolarization. On the electrocardiogram (ECG), as observed in patients with type 2 congenital long QT, IKr inhibition increases the duration of ventricular repolarization (QTc), particularly in the terminal phase (Tpeak-Tend, i.e. TpTe), decreases T wave maximal amplitude (TAmp) and alters T-wave morphology by producing notches [3,4]. Notches corresponds to an additional deflection with inverse polarity during the repolarization phase and is associated, among patients with LQTS, with a further increase of ventricular arrhythmia risk [5,6].

However, the risk of developing drug-induced arrhythmias, prolonged QT and other ECG alterations reflecting IKr inhibition vary markedly between subjects, which suggests the existence of predisposing genetic factors. Congenital LQTS are typically caused by mutations in genes (≈15) related to cardiac ion channels, resulting in impairment of cardiac repolarization [6]. However, the prevalence of these mutations in patients with diLQTS is limited. The current understanding favors the additive effect of common and less common genetic variants that reduces the cardiac repolarization reserve [7–11]. As a consequence, diLQTS can develop among individuals with predisposing genetic factors, which favors exaggerated response to a pharmacological challenge with QT-prolonging drugs [1,11] but does not affect cardiac repolarization features at baseline [1,7,12]. Therefore, the detection of patients prone to develop diLQTS ultimately requires prospective evaluation of changes in cardiac repolarization in response to drug therapy.

For this purpose, we conducted the GENEREPOL study (NCT00773201) where almost 1000 healthy subjects were prospectively challenged with a standardized pharmacological stress (sotalol, 80mg orally). Sotalol is a class III (potassium current inhibitor) anti-arrhythmic drug mainly used to prevent atrial fibrillation, which is associated with one of the highest risks of developing diTDP [8]. Sotalol has linear pharmacokinetics, an almost complete absorption with little variability and a maximal concentration expected 3 hours post oral intake [13]. This allowed for evaluation of IKr inhibition by ECG 3 hours post sotalol dosing with reduced pharmacokinetic variability. Parameters (QTc and TpTe prolongation, TAmp decrease and appearance of notches) were compared at baseline and 3 hours post-dosing to assess the amplitude of IKr inhibition between individuals. We then conducted a genome wide association study (GWAS) to test for common single nucleotide polymorphisms (SNPs) associated with inter-individual sensitivity to IKr inhibition evaluated by ECG surrogates.

## Methods

### Design of the study

**Fig 1:**
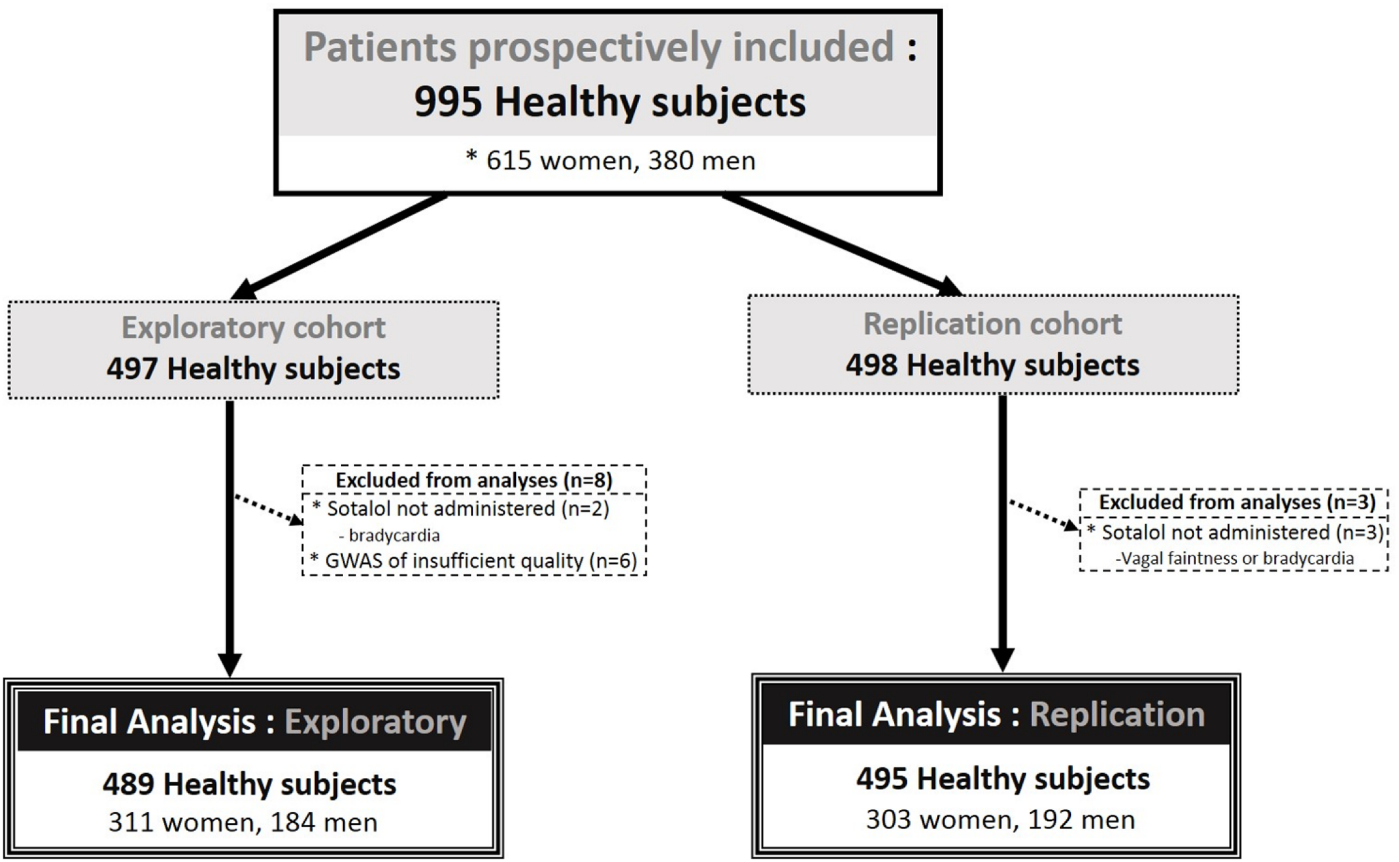
Flow chart of the study.

The GENEREPOL study (clinical trials.gov: NCT00773201) was an open-label prospective study where healthy volunteers were challenged with 80 mg sotalol oral intake to perform GWAS for genetic factors involved in variation of IKr inhibition evaluated on the ECG. From February 2008 to February 2012, 995 healthy volunteers were enrolled in this study (Fig 1). Time of participation in this study was one day. Inclusion criteria were male or female, aged between 18 and 60 years, only of European or North African origin, with a body mass index between 19 and 29 kg/m^2^ and able to give an informed consent. Exclusion Criteria were pregnancy, asthma, resting heart rate below 50 bpm, abnormal ECG (including right bundle branch block) or QRS>100msec, systolic blood pressure<100 mmHg, history of atrioventricular block or Raynaud phenomenon, known chronic illness such as cardiac or renal insufficiency with chronic treatment, QT prolonging drug or any chronic treatment except for contraceptive pills, antalgics and vitamins, family or personal history of congenital long QT syndrome, arrhythmia or sudden death and QTc Fridericia(QTcF)>450ms. The study protocol and all methods applied were approved by the Committee for the Protection of Human Subjects of Paris Ile de France V (Paris, France) and prior written informed consent was obtained from all subjects after being fully informed regarding the nature and risks of the study. We did the study in accordance with the principles of the international conference on harmonization guidelines on good clinical practice and the world medical association declaration of Helsinki.

Volunteers were hospitalized at 8:00am for a duration of approximatively 6 hours at the Clinical Investigation Centre Paris-Est (Saint-Antoine and the Pitié-Salpêtrière Hospital, Paris, France) after an overnight fast. An intravenous catheter was inserted for blood collections; then, a continuous digital 12-lead ECG recording monitoring was started using a Cardioplug device (Cardionics Inc, Brussels, Belgium) connected to a personal computer. Baseline ECG recordings (triplicate of 10-seconds each) were obtained after the subjects had rested for at least 10 minutes in the supine position. Each subject was then given a single oral dose of sotalol (80mg) and ECG monitoring was continuously pursued. Three hours post dosing (H3), 10-second ECG recordings were again extracted (triplicate) after the subjects had rested for at least 10 minutes in the supine position before lunch. At H3, a blood sample was drawn for the determination of plasma sotalol concentration. The participants were finally discharged 5 to 6 hours post dosing after verifying that their QTcF was < QTcF baseline + 40 msec.

### Biological and DNA evaluation

DNA samples from all GENEREPOL participants were extracted from peripheral blood leukocytes (on 5ml blood) using the Puregene extraction kit. All DNAs were tested by nanodrop ensuring high quality in terms of purity (average 260/280 ratio: 1.89).

Sotalol was quantified in plasma (5 ml of lithium heparinate) using a High Performance Liquid Chromatography (Laboratoire de Biochimie Pharmacologie et Toxicologie, Faculté de Médecine Paris-Ile-de-France-Ouest—Université de Versailles Saint-Quentin, CHU Raymond Poincaré, Garches, France).

### Electrocardiographic phenotyping evaluation

As diLQT induced by sotalol represents the equivalent of a iatrogenic form of congenital LQT2 syndrome [3,4], we evaluated the apparition of its classical ECG features between baseline and three hours post sotalol (H3). QTcF (S1 Fig); TpTe, TAmp (S2 Fig) and presence of notch were carefully quantified. Fridericia’s correction (QT/RR^0.33^) was chosen for QT correction on heart rate according to ICH E14 Guideline [14]. In our cohort, after verification, QTcF provided an accurate correction of QT on heart rate. Analysis of all ECGs was performed after inclusion of all the subjects using CARDIABASE post-treatment software (Banook Group, Nancy, France, S1 and S2 Figs).

Analyses of quantitative traits, QTcF, TpTe and Tamp, was performed by two expert investigators trained altogether for these analyses. TpTe was measured by the tangent method in triplicate on a representative beat (10 sec ECG) in leads V3, V4, and V5. Tpeak was positioned at the place of first peak, even if T wave amplitude was not maximal at this point due to notching. The mean value of the three latter leads of a triplicate evaluation was retained (S2 Fig) [15–17]. When measurement of TpTe was impossible in V3, V4 or V5, leads V2 (for V3) or V6 (for V4/V5) were used in substitution. TAmp was positioned at the place of first T wave maximal amplitude on a representative beat (10 sec ECG) in leads LII, V2, and V3. The mean TAmp value of the three latter leads of a triplicate evaluation was retained (S2 Fig) [18–20]. If measurement of one TAmp was impossible in LII, V2 or V3, mainly because of low voltage of T-wave maximal amplitude (<0.1mV), lead V4 was used in substitution. QTcF was measured by the tangent method in LII on three consecutive beats and the mean value of a triplicate evaluation was retained (S1 Fig) [21–22]. Inter- and intra-observer agreements were periodically assessed using ICC (intra-class correlation coefficient) which was continuously measured >0.9, indicating an excellent agreement (hence interchangeability) and repeatability between operators.

For quantitative parameters, the change (Δ) between baseline and the value at H3 was then calculated as follows:

$$\Delta \text{ QTcF }\left(\%\right)=\left(\frac{H3\;Mean\;QTcF\;-\;Baseline\;Mean\;QTcF}{Baseline\;Mean\;QTcF}\right)*100$$ (1)

$$\Delta \text{ TpTe }\left(\%\right)\;=\left(\frac{H3\;Mean\;TpTe\;-\;Baseline\;Mean\;TpTe}{Baseline\;Mean\;TpTe}\right)*100$$ (2)

$$\Delta \text{ TAmp }\left(\%\right)=\left(\frac{Baseline\;Mean\;TAmp\;-\;H3Mean\;TAmp}{Baseline\;Mean\;QTcF}\right)*100$$ (3)

**Fig 2:**
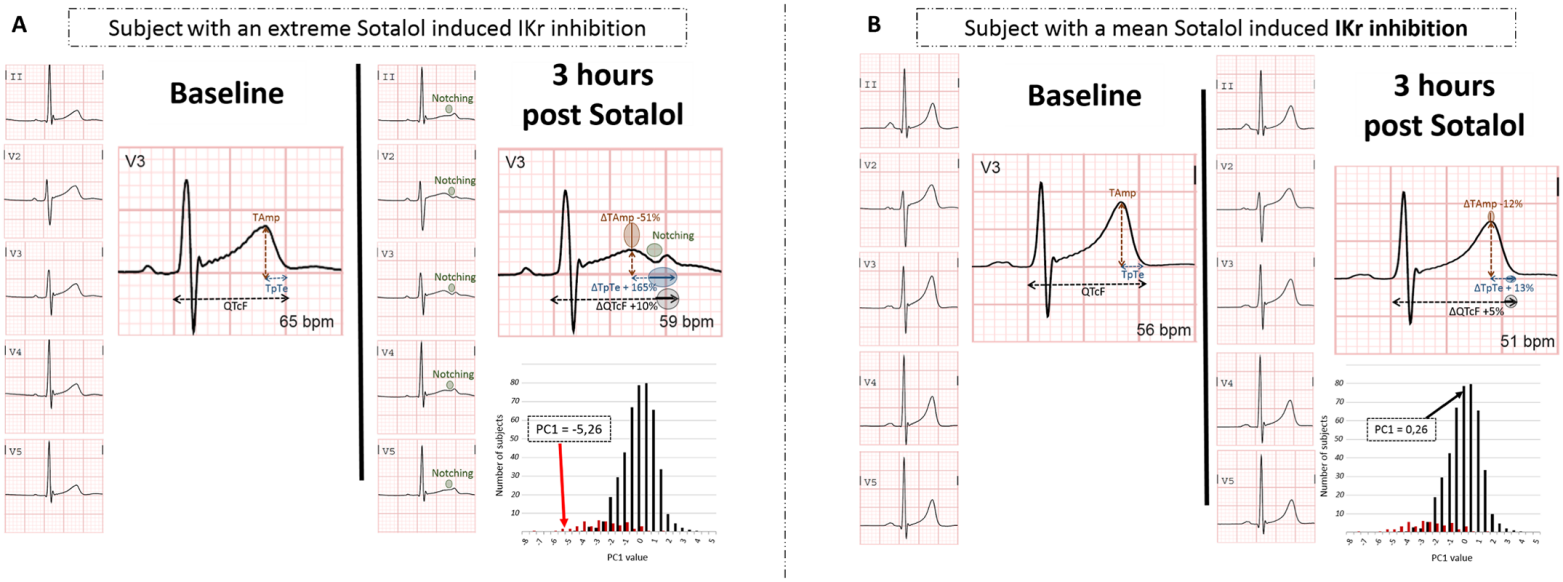
Typical QT and T-wave changes (A) in a subject with a pronounced sotalol-induced IKr inhibition indicated by a notch and (B) in a subject with minimal sotalol-induced IKr inhibition. The PC1 values and distribution of discovery cohort are shown. PC1 is issued from principal component analysis of ΔQTc, ΔTpTe and ΔTAmp. “Notcher” subjects are represented in red and “non notcher” in black.

For qualitative notching evaluation, all ECG were analyzed by JES and CFB. A subject was considered as “notcher” (Fig 2A) or not “notcher” (Fig 2B) when both evaluators separately agreed on the classification. In case of discordance, the subjects were not included in any of these latter groups (corresponding to n = 10 in discovery and n = 8 in replications cohorts).

### Genotyping and Imputation

Within the discovery set of individuals, DNA samples were genotyped with the Illumina-Human610 Quad-beadchip. SNPs with genotyping call rate <99%, minor allele frequencies (MAF) <1%, or showing statistical significant (p<10^−5^) deviation from Hardy-Weinberg equilibrium were filtered out. The application of these filters led to the selection of 505,432 autosomal SNPs with validated quality-controls. Individuals were excluded in presence of a genotyping rate lower than 90% or of close relatedness as suspected by pairwise clustering of identity by state distances and multidimensional scaling as implemented in PLINK. Finally, 489 individuals were left for imputation and association analysis in the discovery set.

The 505,432 QC-checked SNPs were then used for imputing 40,309,712 autosomal SNPs from the 1000 Genomes 2012–02 release CEU reference dataset. For this, the MACH (version v1.0.18.c) software was used. All SNPs with acceptable imputation quality (r^2^>0.4) and estimated MAF>0.01 were kept for association analysis with ECG phenotypes.

SNPs selected from the discovery scan for further validation were first genotyped using allele-specific PCR in the discovery samples to assess the validity of the imputation. SNPs with consistent imputation and wet-lab genotyping results were further genotyped in the replication sample to confirm their association with the studied ECG phenotypes. Allele-specific PCR genotyping was outsourced at LGC genomics (Herts, UK).

### Descriptive statistical data analysis

Demographic and ECG data were described by standard descriptive statistics as mean ± standard deviations or median and interquartile range, where appropriate. Comparison of quantitative variables were analyzed by Student’s t-test, Mann-Whitney tests, Kruskal-Wallis tests or ANOVA and Tukey’s post-test as appropriate. Comparison of qualitative variables were performed by Chi-2 test. The normal quantile transformation [23] was applied to the ΔTpTe variable to make its distribution Gaussian before GWAS analyses. A principal component analysis was applied to the three ΔQTcF, ΔTpTe and ΔTAmp phenotypes to identify surrogates markers (further ventricular repolarization phenotyping) [24] that better integrate the information brought by the three correlated Δ values. All statistical analyses were performed using the R package.

### Genome-wide association analysis with ECG phenotypes in the discovery cohort

Association of imputed SNPs with the three ECG Δ phenotypes (and their derived principal components) was assessed by a linear regression analysis as implemented in the MACH2QTL (version1.1.2) software [25]. The MACH2DAT (version1.0.19) software implementing a logistic regression model was employed to assess the association of imputed SNPs with notches apparition. In all these analyses, the allele dosages representing the expected numbers of a given reference allele at the imputed SNPs were used to model the influence on the phenotype of the tested SNP. Association analyses were adjusted for age, sex, basal kalemia, ethnic origin, sotalol plasma levels at 3hours, and the first 5 principal components (PCs) derived from the analysis of the genotyped SNPs.

A statistical threshold of 5x10^-8^ was applied to declare genome-wide statistical significance. Any genome-wide significant SNP was further wet-lab re-genotyped in the discovery sample to assess the validity of the imputation inference. All SNPs with consistent imputation results and wet-lab genotyping were then pursued for replication in the validation sample.

### Association analysis in the validation sample

The same phenotype transformations as in the discovery cohort were applied in the validation sample. Association of tested SNPs with studied phenotypes was also assessed by means of linear/logistic regression analysis under the assumption of additive allele effects adjusting for the same confounding variables as in the discovery cohort except the genetic PCs.

For all genotyped SNPs in the discovery and validation samples, deviation from Hardy-Weinberg equilibrium was tested by a Chi-square test statistic with one degree of freedom.

## Results

### Population characteristics

A total of 995 healthy subjects were prospectively included in the study, and of them, 497 participated in the discovery cohort. Eight subjects were excluded because of missed sotalol administration or insufficient quality of genome-wide genotyping leaving a total of 489 subjects for GWAS. The remaining 498 individuals participated in the replication cohort of whom 495 actually received sotalol and were kept for analysis (Fig 1). Main clinical, biological and electrocardiographic variables in the discovery and replication cohorts are reported in Table 1. The majority of subjects were of European ancestry and females. Baseline biological and electrocardiographic parameters were within the normal range as anticipated in these healthy subjects: kalemia (4.0±0.3 vs. 4.1±0.3 mmol/L, p<0.001), mean QTcf (389±17 vs. 387±17msec, p = 0.02) and maximum QTcF (432 vs. 439 msec) at baseline in discovery and replication cohort, respectively.

**Table 1:** Demographic and baseline ECG characteristics of patients who received sotalol.

	Discovery cohort	Replication cohort	Pvalue
**Subjects who received sotalol**	495	495	ns
**Age** (years)	29±10.8	28±10.2	ns
**Female** (%)	311(63%)	303(61%)	ns
**Kalemia** (mmol/l)	4±0.3	4.1±0.3	<0.001
**Plasma sotalol at H3** (ng/ml)	398±134	448±153	<0.001
**European / North-African**	448 (90%)/47(10%)	431(87%) / 64(13%)	ns
**Baseline QTcF** (mean±SD), msec	389±17	387±17	0.02
**Baseline QTcF** (median,[IQR]), msec	388 [374; 398]	389 [377; 399]	0.03
**Baseline QTcF** (Min; Max), msec	333; 432	333; 439	
**Baseline RR** (mean±SD), msec	905±115	907±102	ns
**Baseline TAmp** (mean±SD), mV	512±177	531±183	ns
**Baseline TpTe** (mean±SD), msec	70±9	67±9	<0.001
**Baseline Notching** (%)	0(0%)	0(0%)	ns

ns represents p>0.05. Mean ± standard deviation and median [interquartile range] are shown.

### Sotalol-induced electrocardiographic changes

**Fig 3:**
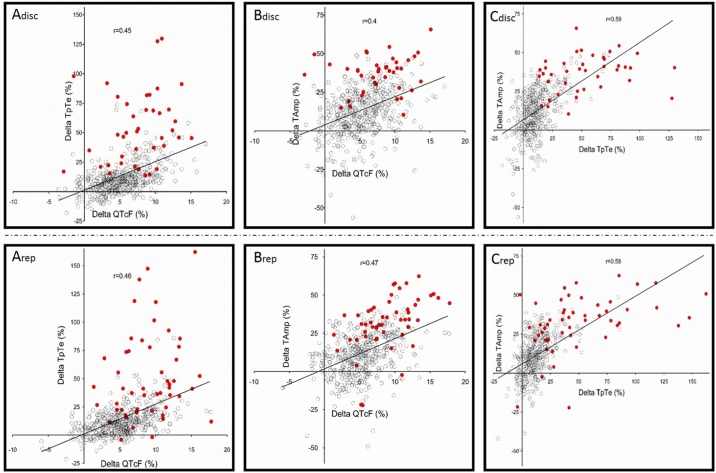
Correlations between ΔQTcF (%), ΔTpTe (%) and ΔTAmp (%) in the discovery (Adisc-Cdisc, n = 489) and replication cohort (Arep-Crep, n = 495). “Notcher” subjects are represented in red.

Three hours post sotalol administration, electrocardiographic changes suggested scattered IKr inhibition and were similar among the discovery and replication cohorts for all measured quantitative and qualitative parameters (Tables 1 and 2). In line with previous studies, 80mg oral sotalol induced absolute and relative changes in the length of QTcF of 21.4±14ms and 5.5±3.5%, respectively. Typical changes in the T-wave morphology were also observed in the discovery and replication cohorts with an increase in TpTe (14.2±15.6 and 15.9±20.5%, respectively; p = ns) and a decrease in TAmp (13.6±15.7 and 12.8±15.3%, respectively; p = ns). However, there was large inter-individual variability in all ECG changes as shown in Table 2 and Fig 3. Variations in TpTe, QTcF and TAmp between H3 and baseline were auto-correlated in the same proportion of subjects among the two cohorts (Table 3 and Fig 3). Finally, 40 (8%) and 51 subjects (10%) respectively presented with a typical notch in each cohort. Subjects presenting with notches (Fig 2) were quasi-exclusively women and had higher ΔTpTe, ΔQTcF and ΔTAmp than non notchers (Table 4).

**Table 2:** ECG characteristics of patients 3 hours post sotalol administration.

	Discovery cohort (n = 495)	Replication cohort (n = 495)	Pvalue
**Individual measures**			
QTcF H3, mean (SD), msec	410 (24)	408 (24)	ns
QTcF H3, median (IQR), msec	408 [394; 425]	408 [391; 424]	ns
QTcF H3, Min; Max	331; 499	329; 477	
QTcF H3, > 450 msec	23(4.6%)	31(6.3%)	ns
RR H3, mean (SD), msec	1070 (132)	1067 (130)	ns
TAmp H3, mean (SD), mV	451 (194)	472 (203)	ns
TpTe H3, mean (SD), msec	79 (17)	78 (18)	ns
Notching (%)	40(8%)	51(10%)	ns
**Changes as compared to baseline**			
**Absolute ΔQTcF**, msec			
mean (SD)	21 (14)	22 (14)	ns
Median (IQR)	20 [11.7; 30]	21 [12; 31]	ns
Min; Max	-13.7; 73	-23; 72	ns
> 50 msec	12(2.4%)	15(3%)	ns
**Relative ΔQTcF, %**			
mean (SD)	5.4 (3.5)	5.6 (3.6)	ns
Median (IQR)	5.2 [3; 7.5]	5.4 [3.2; 7.8]	ns
Min; Max	-5.7; 17.1	-6; 17.8	ns
**Relative ΔTamp, %**			
mean (SD)	13.6±15.7	12.8±15.3	ns
Median (IQR)	10 [4; 17.5]	10 [4; 17.5]	ns
Min; Max	-56.3; 65.7	-48.9; 62.4	ns
**Relative ΔTpTe, %**			
mean (SD)	14.2±15.6	15.9±20.5	ns
Median (IQR)	13.3 [3.2; 23.9]	13.3 [3.2; 23.9]	ns
Min; Max	-18.1; 129.8	-21.3; 162.2	ns

ns represents p>0.05. Mean ± standard deviation and median [interquartile range] are shown.

**Table 3:** Correlations (r) between ΔTAmp, ΔTpTe, ΔQTcF and results of principal component (PC) analysis in discovery (n = 489, in bold) and replication (n = 495, highlighted in italic) cohort.

**Correlation (r)**	ΔTAmp	ΔTpTe	ΔQTcF	PC1	PC2	PC3
ΔTAmp	1	**0.59**	**0.40**	**-0.83**	**0.36**	**-0.43**
ΔTpTe	*0*.*58*	1	**0.45**	**-0.85**	**0.23**	**0.47**
ΔQTcF	*0*.*47*	*0*.*46*	1	**-0.73**	**-0.68**	**-0.06**
PC1	*-0*.*84*	*-0*.*84*	*-0*.*78*	1	**0**	**0**
PC2	*0*.*28*	*0*.*3*	*-0*.*63*	*0*	1	**0**
PC3	*-0*.*46*	*0*.*45*	*0*.*01*	*0*	*0*	1

**Table 4:** Electrocardiographic findings of patients who presented or did not present with notching 3 hours post sotalol administration in the discovery and replication cohort.

	Discovery cohort		Replication cohort		Pvalue
	Notching	No Notch	Notching	No Notch	
**Number of subjects**	40	445	51	436	
**Female** (%)	40(100%)	263(59%)	50(98%)	247(57%)	<0.0001
**ΔQTcF** (%)	7.7±4^†^	5.1±3.3^‡^	9±3.7^†^	5.2±3.4^‡^	<0.0001
**ΔTAmp** (%)	36.7±11.9^†^	11.3±14.2^‡^	32.1±17.5^†^	10.4±13.4^‡^	<0.0001
**ΔTpTe** (%)	53.6±30^†^	10.4±11.8^‡^	51±38.4^†^	11.7±12.1^‡^	<0.0001
**Principal Component 1**	-2.5±1.5^†^	0.3±1.1^‡^	-2.3±1.8^†^	0.3±1.1^‡^	<0.0001
**Principal Component 2**	0.7±1.1 ^†^	-0.1±0.7 ^‡^	0.3±1 ^†^	0±0.7 ^‡^	<0.0001
**Principal Component 3**	0.5±1.2 ^†^	0±0.5 ^‡^	0.3±1.3 ^†^	0±0.5 ^‡^	<0.0001

Pvalue: One-way Anova with Tukey’s post-test used.

^†^: Significant vs. No notch groups (discovery and validation cohort);

^‡^: Significant vs. notching groups (discovery and validation cohort).

### GWAS results

8,306,856 autosomal imputed SNPs were tested for association with the studied ECG phenotypes. The Manhattan and Quantile-Quantile plots summarizing the results of these GWAS analyses are shown in S3 Fig. No association signal reached the advocated genome-wide significance level of 5x10^-8^ for ΔQTcF, ΔTAmp and normalized ΔTpTe phenotypes. The strongest associations were observed at *ANXA5* rs2621223 (p = 1.49x10^-06^) for ΔQTcF, *SHISA9* rs79922451 (p = 3.85x10^-7^) for ΔTpTe, and *LRRC4C* rs74746008 (p = 6.25x10^-8^) for ΔTAmp. Association of imputed SNPs with notches apparition was only performed among women (n = 298) since no males were reported as notcher in the discovery cohort. The strongest association was observed at *PRR5L* rs3812774 (p = 2.64x10^-7^) for notching. However, after validation of the genotypes for these candidates in both cohorts, these associations were not significant in the validation cohort, suggesting the lack of influence of common variants on these typical ECG parameters.

### Principal component analysis

To increase the sensitivity of our discovery phase, we applied the GWAS framework to the three principal components variables derived from the ΔQTcf, ΔTpTe and ΔTAmp phenotypes, following the recent suggestion by Aschard et al. [26]. Principal component (PC) analysis generated three quantitative parameters related to ventricular repolarization modification induced by sotalol. The first principal component (PC1) that explained most (~65%) of the total phenotypic variance reflected the typical changes after IKr inhibition, i.e a joint increase of TpTe, QTcF and decrease in TAmp (Table 3). We found significant decreases in PC1 values (p<10^−4^) in notchers as compared to non notchers in both discovery (-2.5±1.5 vs. 0.3±1.1) and replication (-2.3±1.8 vs. 0.3±1.1) cohorts, with consistent results only when restricting to female subjects (-2.5±1.5 vs. -0.2±1.1 in discovery and -2.3±1.8 vs. -0.2±1 in replication, p<10^−4^). These results further confirmed PC1 as a valuable quantitative surrogate of IKr inhibition (Table 3). Distribution of PC1 values are shown in Fig 2 with an example of one subject with an extreme negative PC1 value and pronounced IKr inhibition with notching. In the replication cohort, the percentage of phenotypic variance explained by this corresponding first principal component was concordant (~67%). The second (PC2) and third (PC3) components explained a smaller proportion of the total phenotypic variance in the discovery samples, 21% and 14%, respectively. Corresponding values in the replication cohort were 19% and 14%, respectively. PC2 and PC3 were associated with different phenotypic patterns among both cohorts regarding the effects of sotalol on ventricular repolarization (Table 3).

We then applied the GWAS framework to the three principal components (PC1-PC3) variables. No genome-wide significant associations were observed for PC1 and PC2. Five loci were found significantly associated with the variability of PC3 (Table 5). After having validated the genotypes of the most significant SNPs at each of these loci in the discovery cohort (*SEL1L* rs61986295, *SP110* rs146598419, *PLA2G5* rs7796806, *LINC003* rs117161099 and *PGR* rs76176654), these SNPs were followed up in the replication cohort for further validation. However, these associations were not significant in the replication cohort (Table 5). The Manhattan and Quantile-Quantile plots summarizing the results of these GWAS analyses applied on principal components variables are given in S3 Fig.

**Table 5:** SNPs significantly associated at p<5*10^−8^ and imputation r^2^>0.4 with an ECG phenotype in the discovery imputation association analysis and their validation in an independent sample.

				Discovery cohort					Replication cohort		
Gene	rs number	Coded allele	Non coded allele	Imputed CAF	β (se)	Pvalue	Phenotype	Observed CAF	Observed CAF	β (se)	Pvalue
***SEL1L***	rs61986295	T	C	0.011	1.504 (0.25)	1.65_*_10^−10^	PC3	0.012	0.012	0.148 (0.17)	0.4
***SP110***	rs146598419	C	T	0.012	1.311 (0.21)	3.99_*_10^−10^	PC3	0.003	0.001	0.520 (0.65)	0.42
***PLA2G2A***	rs7796806	A	C	0.04	0.635 (0.11)	1.46_*_10^−9^	PC3	0.036	0.036	0.076 (0.12)	0.51
***LINC00301***	rs117161099	T	C	0.012	1.315 (0.24)	2.40_*_10^−8^	PC3	0.006	0.004	-0.644 (0.32)	0.048
***PGR***	rs76176654	T	C	0.013	1.27 (0.23)	4.47_*_10^−8^	PC3	0.010	0.009	-0.141 (0.22)	0.52

Abbreviations: CAF: Coded Allele Frequency, β (se): effect (standard error) associated with the coded allele

No SNP reached statistical significance set at p<5*10^−8^ in the discovery cohort when GWAS was applied to ΔQTcF, normalized ΔTpTe, ΔTAmp (n = 489) and notching in women (n = 298). To increase the sensibility of our discovery phase, we applied the GWAS framework to the three principal components variables derived from ΔQTcf, ΔTpTe and ΔTAmp phenotypes. In the discovery samples, the first principal component (PC) derived from the 3 Δvalues explained 65% of the total phenotypic variance (PC1), the second 21% (PC2) and the last one 14% (PC3). Corresponding values in the replication samples were consistent (67%, 19% and 14%, respectively).

## Discussion

The results of the present study show that: 1/ the administration of a single oral dose of 80 mg of sotalol in healthy subjects reproduces ECG changes that are typically observed in type 2 congenital LQTS; 2/ there are no common SNPs with extreme effects on the individual variability in IKr inhibition induced by sotalol; 3/ a principal component analysis based on ΔQTcF, ΔTAmp and ΔTpTe might be an integrative way to further differentiate patients with the most extreme IKr inhibition.

Several relevant clinical risk factors (such as recent conversion from AF to normal rhythm, bradycardia, hypokalemia, female gender or steroid hormones levels) have been associated with the risk to develop diLQTS or iatrogenic Torsades de pointes [1,12,27–29]. However, individual predictability remains low and it has been suggested that genetic variants might explain a significant proportion of susceptibility to develop diLQTS. This is supported by the similarity with the congenital forms of LQTS, which has been found to be associated with multiple rare mutations in 15 genes [30]. Secondly, QT interval responses to a pharmacological stress are exaggerated among first-degree relatives of patients who developed diTdP [7]. Yet, the genetic make-up favoring the occurrence of diLQTS remains undetermined. Candidate gene screening studies have been performed to explain diLQTS in large cohorts of patients as a form frustre of congenital LQTS. However, the prevalence of common mutations in the known LQTS genes was limited (from 10 to maximum 30%) [31–34]. As exemplified in our study, many subjects developing profound changes in cardiac repolarization in response to a pharmacological challenge do not present with clinical or electrocardiographic abnormalities at baseline, nor have familial history of syncope or sudden death [31–34]. In a recent study with a Danish population cohort of 7000 subjects, Ghouse et al. found that 26 congenital LQTS-associated variants (mainly affecting ion channels) did not influence QTc interval duration, syncopes or overall mortality [35]. Current knowledge favors the existence of genetic variants that affect the cardiac repolarization reserve, which could lead to an exaggerated response to a pharmacological challenge [11]. In line with this concept, different studies have reported on the influence of new genes determining QTc duration and T-wave morphology in the general population and associated with regulation of ion channels machinery involving channels turnover and dynamic expression at the plasma membrane in large macromolecular complexes, such AKAP9 and NOS1AP [36, 37]. It has therefore been suggested that diLQT might be associated with variants affecting ion channel machinery. However, we were unable to detect any major influence of common SNPs in these genes in our study (S1 Table). Thus, our study was more focused on the research of SNPs which would be valuable as an easy diagnostic tool for the individual prediction of a diLQT when taking a drug at risk. Of course, our negative results do not invalidate the entire body of GWAS studies which found SNPs with effects on baseline QTc and T-wave morphology. Different groups have further proposed genome-wide studies where genetic investigations were performed in patients identified with diLQT or the event drug induced “Torsades de pointes” and in matched drug-exposed control subjects [7,38]. However, with this latter case/control approach, there are potential biases due to differences in comorbidities, such as ischemic disease or heart failure, electrolyte abnormalities, comedications and ethnicities. GWAS did not find common genetic variants with large impact on diLQTS. Our GWAS study, in accordance with Behr et al. [7] also failed to identify common genetic variants associated with variability of drug-induced QT prolongation. Exome sequencing subsequently performed in a subset of the patients studied by Behr et al. only found an increased burden of rare potassium channel (*KCNE1*) and regulatory protein (*ACN9*) variants [39]. Contrasting with our results, some case/control association studies using targeted candidate approaches with SNPs in 1 to 18 genes already known to influence QTcF have been able to show that common variations in *NOS1AP* (rs10800397, rs10800404, rs12734991)[40], *SCN5A* (rs7626962)[41] and *KCNE1* (rs1805128)[42] were associated with a significant increase in the risk of diLQTS or diTDP. These SNPs were not significantly associated to any phenotypic traits in our study. Furthermore, the SCN5A variant allele (Y1102) was linked with the risk of arrhythmias in African-Americans [41]. However, this association has not been reported in Caucasians, nor was it detected in our study. Of note, trans-ethnic analysis bias did not contribute to our negative results. Restricting GWAS study to European ancestral populations, excluding North-Africans did not change our results.

In our study, we proposed a different strategy where healthy volunteers are prospectively challenged with a standardized unique dose of a QT-prolonging drug and then extensively phenotyped with ECG measurements. Investigations in healthy subjects have the advantage of limiting confounders, such as comorbidities and comedications. In addition, we prospectively collected information on anticipated factors of influence on cardiac repolarization (i.e. kalemia, sex, ethnicity and H3 plasma sotalol level) and used these factors to adjust multivariate analyses. We proposed this approach to limit external variability due to non-genetic confounding factors. However, despite having a power of 80% to detect (at the 5x10^-8^ statistical threshold) the additive effect of any SNP explaining at least 8% of the variability of a quantitative trait, we were not able to identify SNPs associated with such an effect. In our study, 80mg oral sotalol induced a mean absolute change in the length of QTcF of 21.4ms. Therefore, we could estimate that 8% of variability corresponds to an additive genetic effect of +12.8msec (with a MAF of 0.05), +9.3msec (MAF:0.10), +7.8msec (MAF:0.15), +6.5msec (MAF:0.25) and +6.1(MAF:0.30). The power of the study was maximal (>90%) to detect an additive genetic effect >10msec with a minor allele frequency of >0.10. In a recessive model, our study was well-powered to detect influences of common genetic variants (MAF>0.05) which might be valuable as diagnostic tool for the individual prediction of an extreme response to the drug, with a clinically relevant drug-induced QT prolongation above 50msec [43]. Our study was not designed or powered to detect smaller effects, particularly for rare SNPs with low minor allele frequencies. Identifying the influence of such SNPs would require a significant increase in sample size. In our study, we prospectively investigated the response to sotalol in approximatively 1000 individuals, which represents an unprecedented effort. Alternative strategies, such as a GWAS meta-analysis or whole-exome [38] or whole-genome sequencing, should now be tested seeking for rare and low frequency variants which might offer new insight about ventricular repolarization biology.

As expected, we found an increase in TpTe, in QTcF and a decrease in Tamp between baseline and H3 ECG. The dose of sotalol used in GENEREPOL altered ventricular repolarization with appropriate inter-individual variability for our GWAS analysis. ΔQTcF, ΔTpTe and ΔTAmp were moderately auto-correlated and provided a different manner to assess IKr inhibition. Thus, we used principal component analysis, as a multi-parametric tool, to generate new vectors (PC1, PC2 and PC3), independent of each other, allowing for analysis of the variation of the three native parameters (ΔQTcF, Δ TpTe, ΔTAmpl) in a same or divergent way. PC1, which explained most of the total variance of the Δdata, was interestingly associated to the “common mechanism” of a QTcF and TpTe lengthening associated to a TAmpl decrease. It is a complementary quantitative and integrative surrogate of drug-induced IKr inhibition [4]. By refining our phenotypic analysis, we have been able to identify a sub-group of subjects who exhibit the most characteristic IKr inhibition changes on the ECG, suggesting the highest risk to develop cardiac arrhythmias with drugs prolonging QT interval. Unfortunately, our GWAS revealed no significant influence of commons SNPs on this integrative parameter. Further genome sequencing studies focusing on these patients have not been performed so far and are likely to provide highly valuable information.

This study was performed to identify common genetic determinants of sotalol-induced effects on repolarization in order to provide potential clues to the genetics of clinical diLQTS or ventricular arrhythmias. Rather than identifying patients with such clinical conditions, which has already led to negative GWAS results [7], nearly 1000 healthy volunteers were exposed to a sotalol challenge. Therefore, this study does not specifically address the question of whether a population that is at high risk for TdP (hypokalemia, bradycardia, prior heart disease) or has developed episodes of documented TdP would have a different genetic profile from this low risk population. It should be emphasized that diTdP often occurs in apparently healthy subjects, such as those included in this study [44], when they are exposed to an IKr blocker. Of note, in our dataset, 27 subjects (2.7%) had a pronounced QTc lengthening above 50 msec despite being given a low and well-tolerated dose of sotalol that is known to carry little or no risk of clinical TdP [45]. This observed proportion of patients with pronounced QTc lengthening is in line with the expected proportion of patients of potential risk of clinical diTDP with higher dose of sotalol [45].

## Conclusion

This study indicates that common genomic variants do not significantly contribute to extreme inter-individual differences in variability of IKr inhibition induced by sotalol in healthy subjects and which would be valuable for the individual prediction of an abnormal response to the drug per se, i.e. in the absence of other proarrhythmic factors. Future exome or genome sequencing studies on rare and low frequency variants are proposed to further investigate this issue.

## Supporting information

S1 Fig Example of QTc measurement on three consecutive beats by the tangent method. (DOCX)

S2 Fig Example of TAmp and TpTe measurement on a representative beat. (DOCX)

S3 Fig Manhattan and Quantile-Quantile plots summarizing the results of GWAS analyses performed in this study (Qualitative phenotypes: Notching; Quantitative phenotypes: ΔQTcF, normalized ΔTpTe, ΔTAmp and their derived principal component analyses). (DOCX)

S1 Table Results of GWAS showing SNP identified in previous studies as being associated with QT duration [36] or T wave morphology [37] selected on ΔQTcF, normalized ΔTpTe, ΔTAmp phenotypes on the overall discovery cohort (n = 489). (XLSX)

S1 Checklist TREND statement checklist for the study. (PDF)

S1 Protocol Study protocol translation. Summary of the trial protocol.
(DOCX)

S2 Protocol Study protocol ethics committee 2007. Full version of the trial protocol.
(PDF)
