# Supplementary material for: GENomE wide analysis of sotalol-induced IKr inhibition during ventricular REPOLarization, “GENEREPOL study”: Lack of common variants with large effect sizes
Source: PLoS One. 2017 Aug 11;12(8):e0181875. doi: 10.1371/journal.pone.0181875 (PMC5553738; doi:10.1371/journal.pone.0181875)
Supplement: S1 Fig — (DOCX) [file pone.0181875.s001.docx]

*Supplemental Figures and Figure Legends:*

***S1 Fig.*** *Example of QTc measurement on three consecutive beats by the tangent method.*


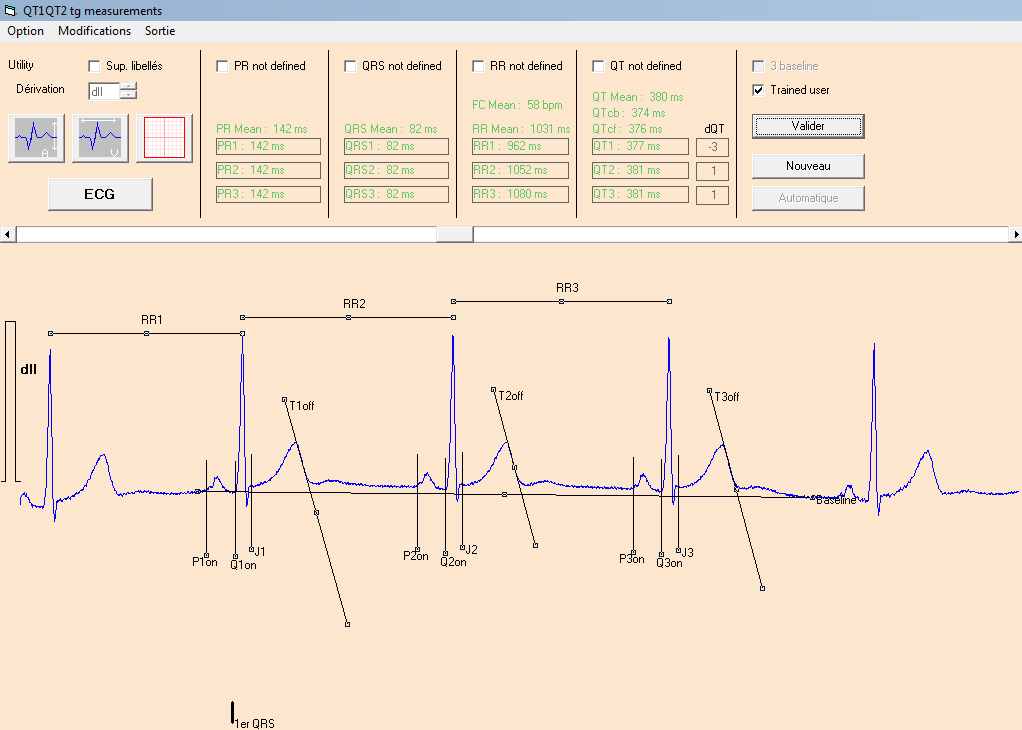


Triplicate : **x 3**
