# Supplementary figures and images for: GENomE wide analysis of sotalol-induced IKr inhibition during ventricular REPOLarization, “GENEREPOL study”: Lack of common variants with large effect sizes
Source: PLoS One. 2017 Aug 11;12(8):e0181875. doi: 10.1371/journal.pone.0181875 (PMC5553738; doi:10.1371/journal.pone.0181875)

***S2 Fig.*** *Example of TAmp and TpTe measurement on a representative beat.*

*
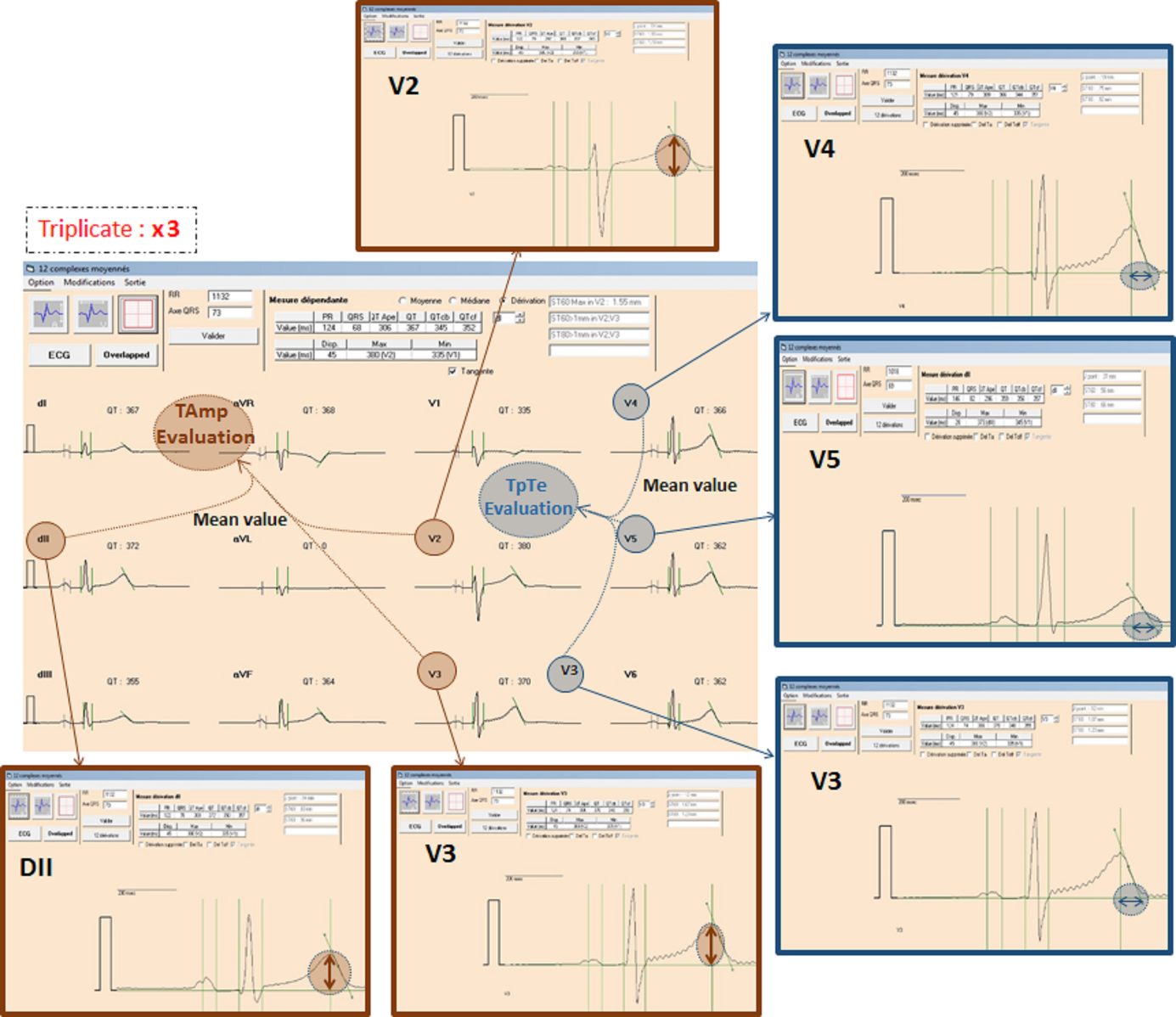
*

Supplement: S2 Fig — (DOCX) [file pone.0181875.s002.docx]
