# Supplementary material for: GENomE wide analysis of sotalol-induced IKr inhibition during ventricular REPOLarization, “GENEREPOL study”: Lack of common variants with large effect sizes
Source: PLoS One. 2017 Aug 11;12(8):e0181875. doi: 10.1371/journal.pone.0181875 (PMC5553738; doi:10.1371/journal.pone.0181875)

***S3 Figs.*** *Manhattan and Quantile-Quantile plots summarizing the results of GWAS analyses performed in this study (Qualitative phenotypes: Notching; Quantitative phenotypes: ΔQTcF, normalized ΔTpTe, ΔTAmp and their derived principal component analyses).*

**GWAS - ΔQT (%)**


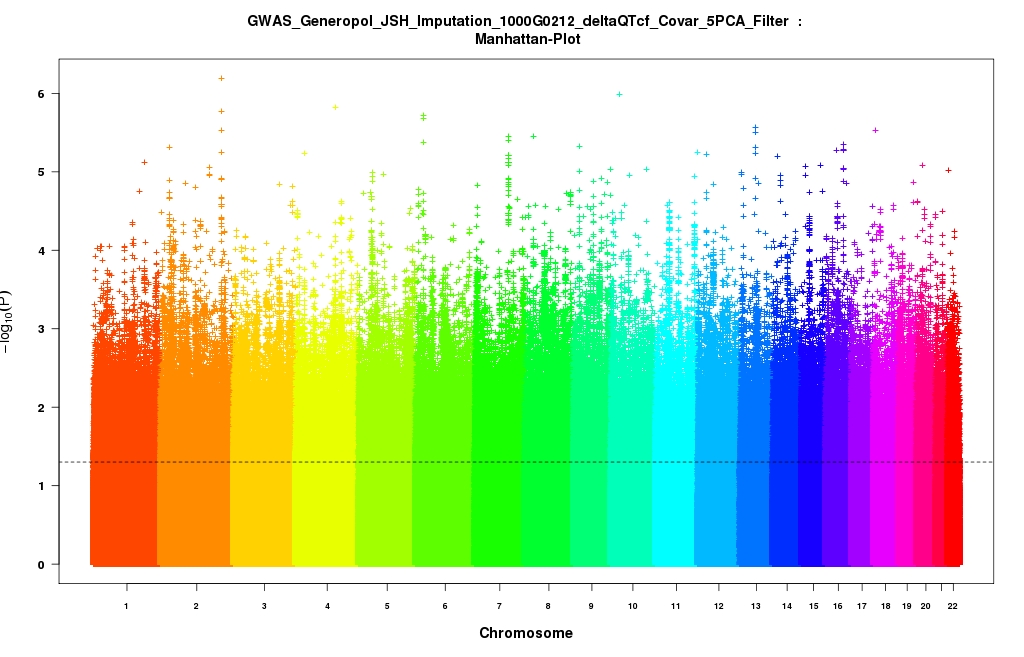


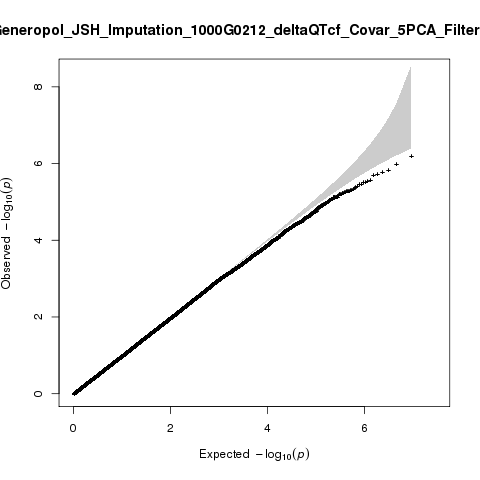


**GWAS - normalized ΔTpTe (%)**


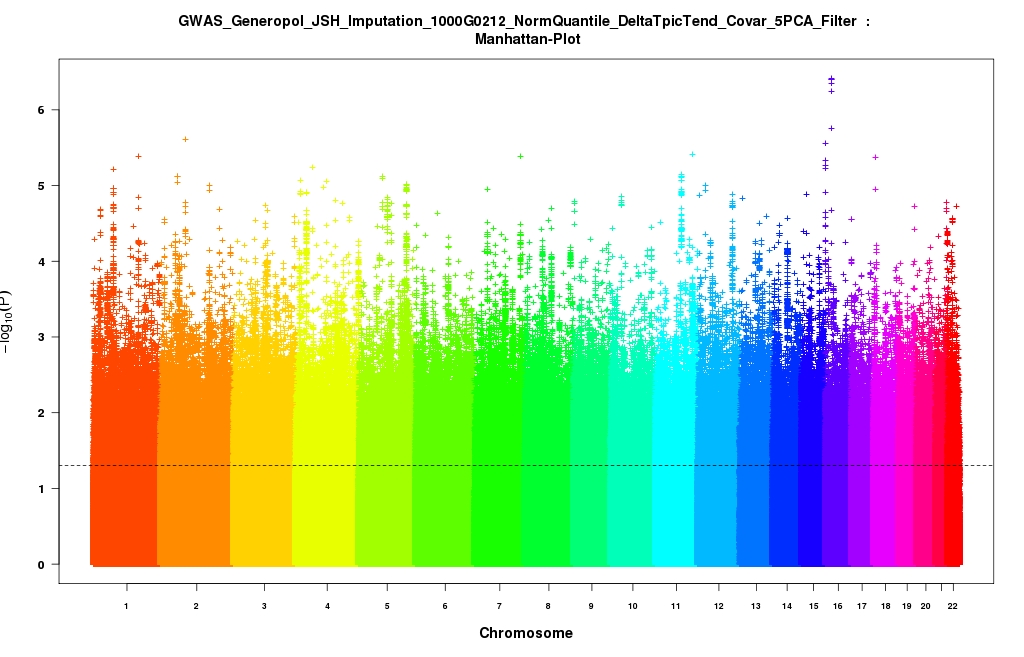


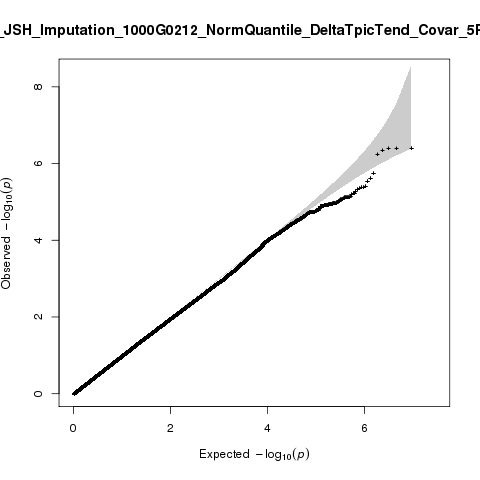


**GWAS - ΔTAmp (%)**


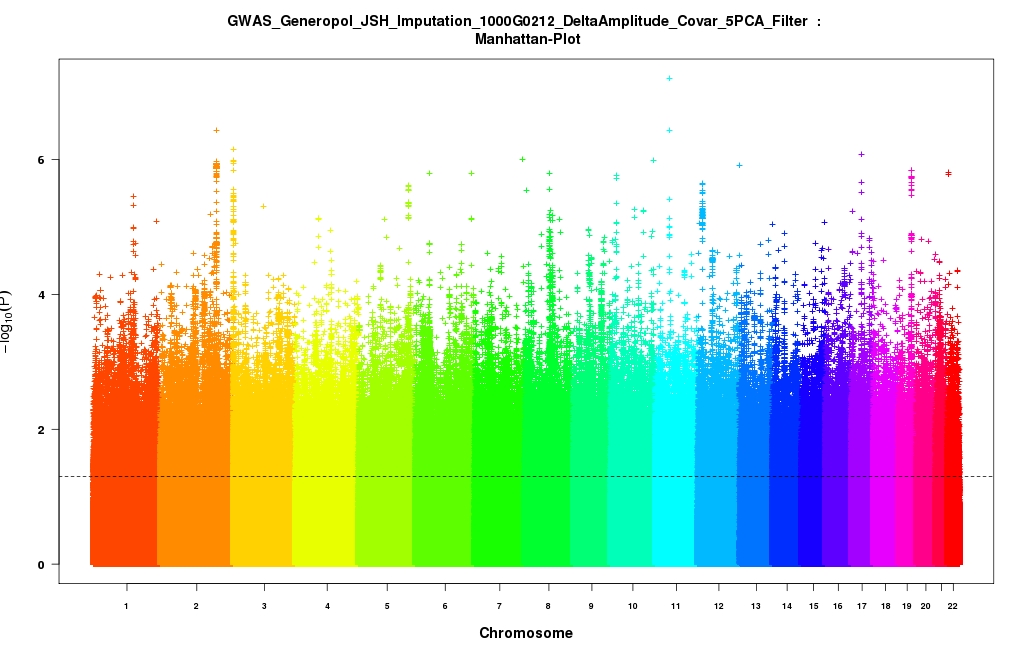


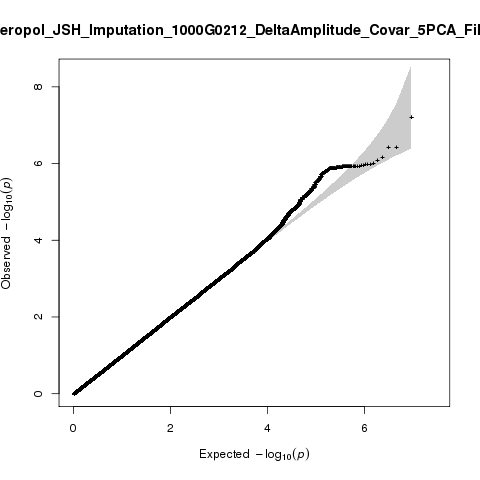


**GWAS – PC1**


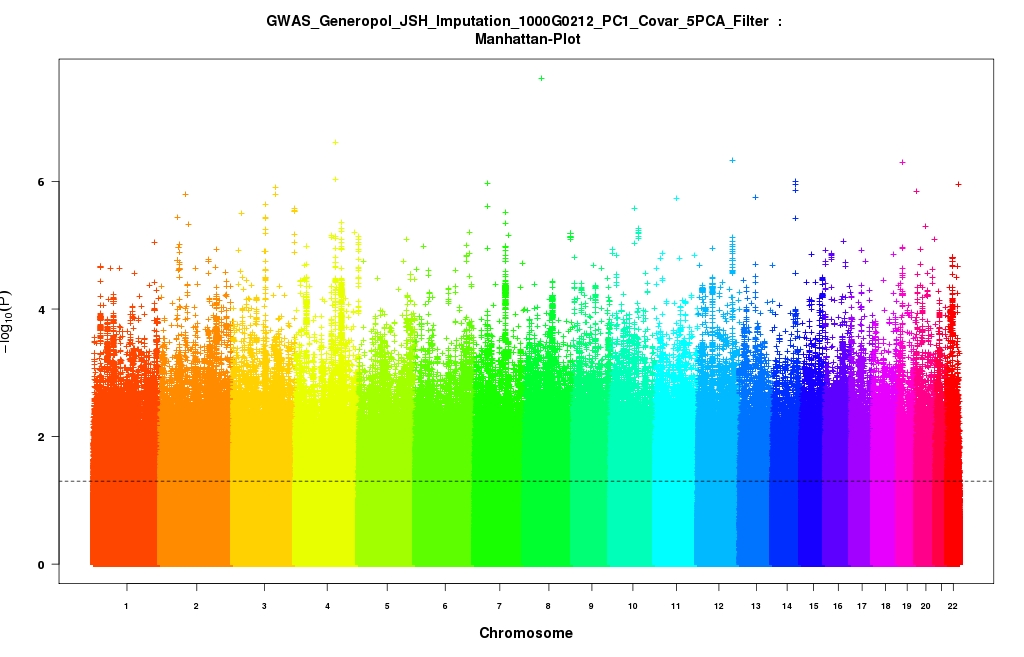


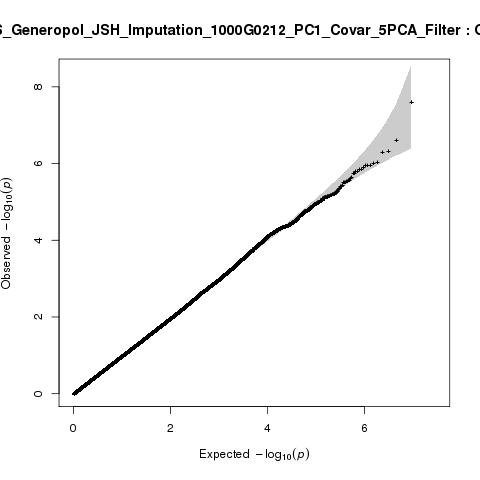


**GWAS – PC2**

**
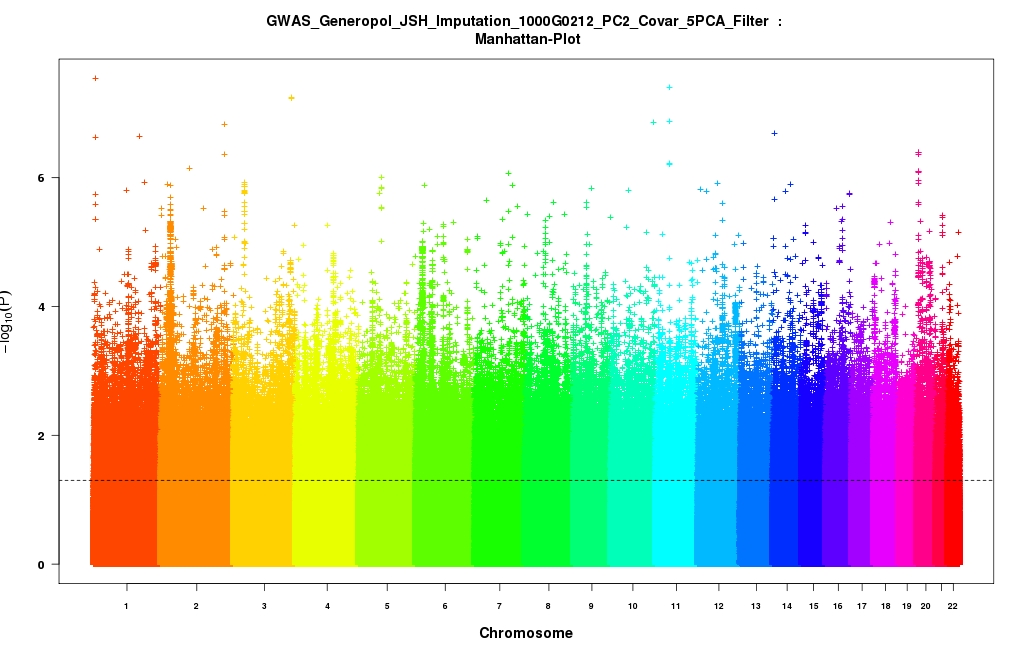
**


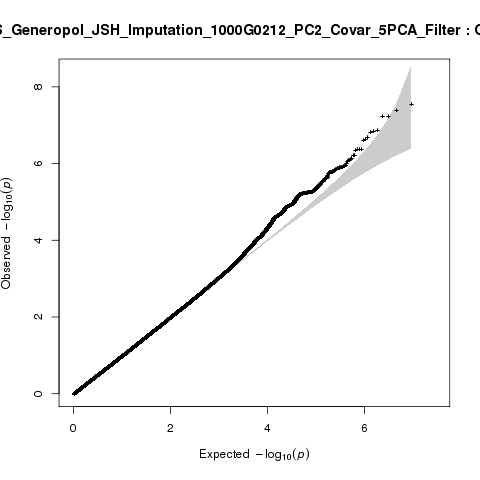


**GWAS – PC3**


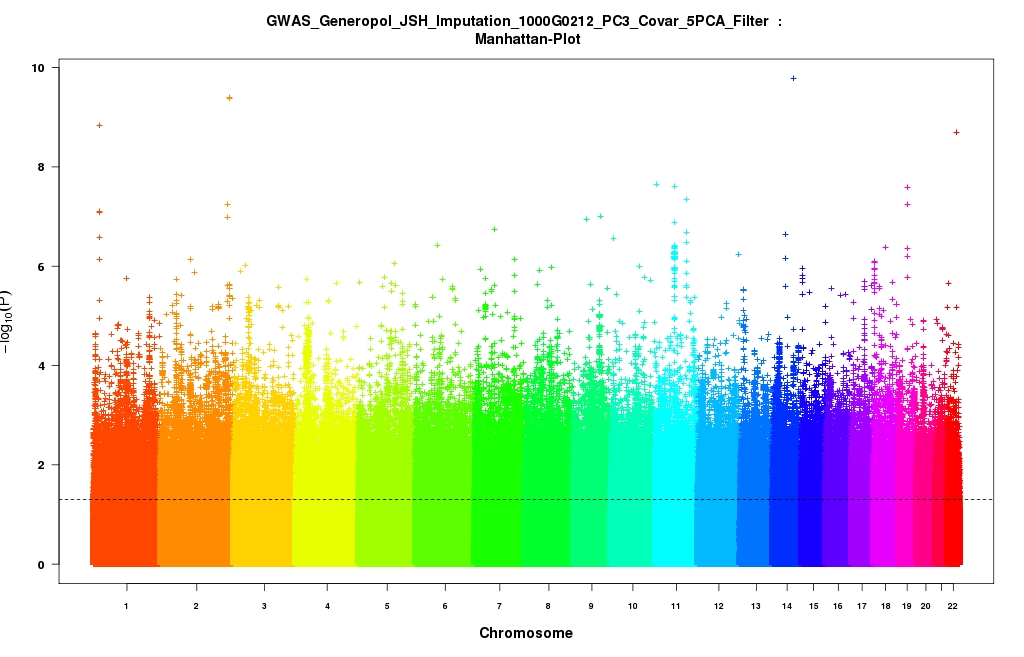


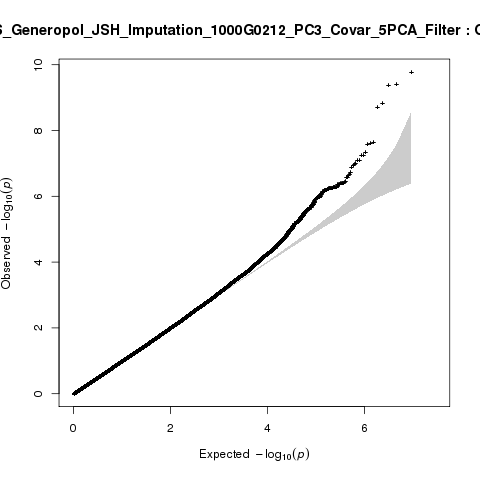


**GWAS – Notch**


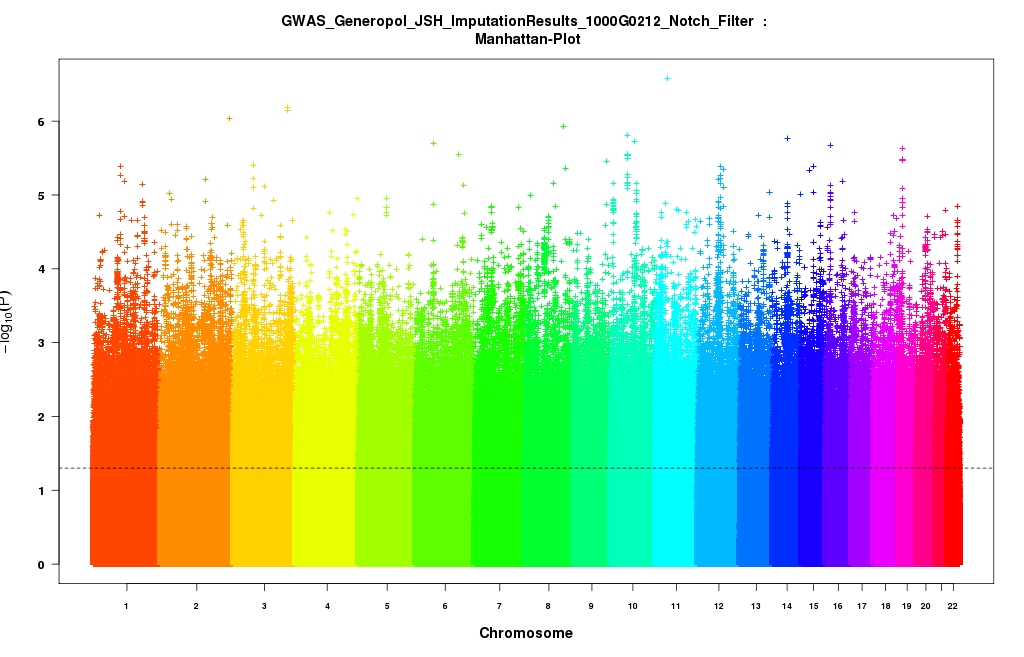


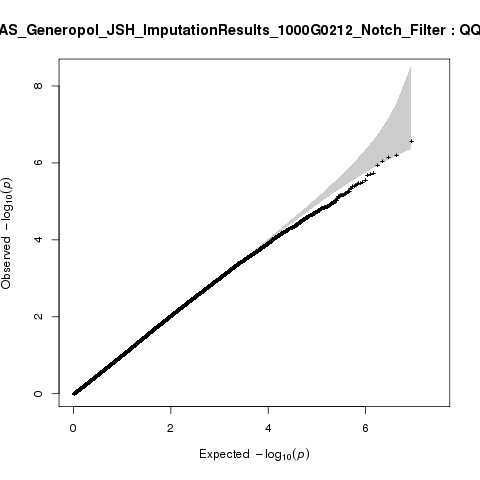

Supplement: S3 Fig — (DOCX) [file pone.0181875.s003.docx]
