# Supplementary material for: GENomE wide analysis of sotalol-induced IKr inhibition during ventricular REPOLarization, “GENEREPOL study”: Lack of common variants with large effect sizes
Source: PLoS One. 2017 Aug 11;12(8):e0181875. doi: 10.1371/journal.pone.0181875 (PMC5553738; doi:10.1371/journal.pone.0181875)
Supplement: S1 Protocol — Summary of the trial protocol. (DOCX) [file pone.0181875.s006.docx]

**GENomE Wide Analysis of Sotalol-induced IKr inhibition during ventricular REPOLarization, “GENEREPOL study”: Lack of common variants with large effect sizes.**

Short title: No common SNP associated to sotalol-induced IKr inhibition

Joe-Elie Salem ^1,2^, Marine Germain ^2^, Jean-Sébastien Hulot ^1,2,^*, Pascal Voiriot ^3^, Bruno Lebourgeois ^1^, Jean Waldura ^3^, David-Alexandre Tregouet ^1^, Beny Charbit ^2,#a^, Christian Funck-Brentano ^1,2^.

^1^ Sorbonne-Universités, UPMC Univ Paris 06, INSERM, UMRS-1166, Institute of Cardio metabolism and Nutrition (ICAN), Paris, France

^2^ AP-HP, CIC-1421-Paris-Est, Pitié-Salpêtrière Hospital, F-75013, Paris, France

^3^ Cardiabase-Banook group, Nancy, France.

^#a^ Present address: CHU Reims, Hôpital Robert Debré, Department of Anesthesiology and Intensive Care, REIMS, F-51092, France

## * Corresponding author:

## E-mail: [jean-sebastien.hulot@aphp.fr](mailto:jean-sebastien.hulot@aphp.fr)

## Pr Jean-Sébastien Hulot; Centre d'Investigation Clinique Paris-Est, Hôpital Pitié-Salpêtrière, Bâtiment Antonin Gosset, 47-83 Bld de l'hôpital, 75651 Paris Cedex 13, Tel: +33 1 42 17 85 31, Fax : +33 1 42 17 85 32;

**Clinical Trial Registration:** <https://clinicaltrials.gov/ct2/show/NCT00773201> , study first received: October 15, 2008

**Journals Subject Terms:** Electrophysiology, Arrhythmias, Basic Science Research, Mechanisms, Pathophysiology, Translational Studies, Cardiovascular Disease, Women, Risk Factors, Genetic, Association Studies, Electrocardiology (ECG), Pharmacology, Treatment

**TRIAL PROTOCOL**

The GENEREPOL study (clinical trials.gov: NCT00773201) was an open-label prospective study where healthy volunteers were challenged with 80 mg sotalol oral intake to perform GWAS for genetic factors involved in variation of IKr inhibition evaluated on the ECG. From February 2008 to February 2012, 995 healthy volunteers were enrolled in this study (Figure 1). Inclusion criteria were male or female, aged between 18 and 60 years, only of European or North African origin, with a body mass index between 19 and 29 kg/m² and able to give an informed consent. Exclusion Criteria were pregnancy, asthma, resting heart rate below 50 bpm, abnormal ECG (including right bundle branch block) or QRS>100msec, systolic blood pressure<100 mmHg, history of atrioventricular block or Raynaud phenomenon, known chronic illness such as cardiac or renal insufficiency with chronic treatment, QT prolonging drug or any chronic treatment except for contraceptive pills, antalgics and vitamins, family or personal history of congenital long QT syndrome, arrhythmia or sudden death and QTc Fridericia(QTcF)>450ms. The study protocol was approved by the Committee for the Protection of Human Subjects of Paris Ile de France V (Paris, France) and prior written informed consent was obtained from all subjects after being fully informed regarding the nature and risks of the study.

Volunteers were hospitalized at 8:00am for a duration of approximatively 6 hours at the Clinical Investigation Centre Paris-Est (Saint-Antoine and the Pitié-Salpêtrière Hospital, Paris, France) after an overnight fast. An intravenous catheter was inserted for blood collections; then, a continuous digital 12-lead ECG recording monitoring was started using a Cardioplug device (Cardionics Inc®, Brussels, Belgium) connected to a personal computer. Baseline ECG recordings (triplicate of 10-seconds each) were obtained after the subjects had rested for at least 10 minutes in the supine position. Each subject was then given a single oral dose of sotalol (80mg) and ECG monitoring was continuously pursued. Three hours post dosing (H3), 10-second ECG recordings were again extracted (triplicate) after the subjects had rested for at least 10 minutes in the supine position before lunch. At H3, a blood sample was drawn for the determination of plasma sotalol concentration. The participants were finally discharged 5 to 6 hours post dosing after verifying that their QTcF was < QTcF baseline + 40 msec.

The main objective was to research for genetic factors involved in the extreme modifications of the QT interval of the electrocardiogram in answer to a pharmacological stimulation (sotalol) and physiological stimulation in the apparently normal general population.

Primary Outcome Measures: The elongation of the corrected interval QT duration 3 hours after the unique oral taking of an 80 mg dose of Sotalol. [ Time Frame: 3 hours after the taking of Sotalol ]

Secondary Outcomes:

- Constitute a biological base and a phenotypic base of resources which will allow to define answer phenotypes to the implemented dynamic tests [ Time Frame: At the inclusion visit ]
- Look for associations between these phenotypes of electrocardiographically answer and mutations or polymorphisms.
