# Supplementary material for: GENomE wide analysis of sotalol-induced IKr inhibition during ventricular REPOLarization, “GENEREPOL study”: Lack of common variants with large effect sizes
Source: PLoS One. 2017 Aug 11;12(8):e0181875. doi: 10.1371/journal.pone.0181875 (PMC5553738; doi:10.1371/journal.pone.0181875)
Supplement: S2 Protocol — Full version of the trial protocol. (PDF) [file pone.0181875.s007.pdf]

ASSISTANCE PUBLIQUE HÔPITAUX DE PARIS

*Délégation Inter Régionale à la Recherche Clinique d'Ile de France*

# « IDENTIFICATION DES SOURCES GENETIQUES DE VARIABILITE DE L'ADAPTATION DE LA REPOLARISATION VENTRICULAIRE A UN STIMULUS PHARMACOLOGIQUE ET PHYSIOLOGIQUE DANS UNE POPULATION APPAREMMENT NORMALE »

Titre abrégé : GENEREPOL

Version n°1 du 16 octobre 2007
AP-HP : P071001 ; Inserm : A07043DS

Promoteur : Assistance Publique – Hôpitaux de Paris (AP-HP)

Investigateur principal
CHARBIT Beny
PHU (mai 2008), Médecin Délégué du CIC St-Antoine
CIC St-Antoine (CIC-9304) et Département de Pharmacologie
Centre d'Investigation Clinique – Service de Pharmacologie
Hôpital St-Antoine
184 rue du Fbg St-Antoine - 75012 – Paris
Tél : 01 49 28 22 03 ; Fax : 01 49 28 21 08 ; Mél : beny.charbit@sat.aphp.fr

Responsable scientifique
HULOT Jean-Sébastien
MCU-PH
INSERM U621 et Département de Pharmacologie
Faculté de Médecine Pierre et Marie Curie – Pitié Salpêtrière
91 Boulevard de l'Hôpital
75634 PARIS CEDEX 13
Tél : 01 40 77 96 49 ; Fax : 01 40 77 96 45 ; Mél : jean-sebastien.hulot@psl.aphp.fr

Chef de projet DRCD
Mr Yannick VACHER
D.R.C.D. Hôpital Saint Louis, 1 av Claude Vellefaux, 75010 Paris
Tél : 01.44.84.17.30, Fax : 01.44.84.17.99

Co-Investigateurs

Pr Christian FUNCK-BRENTANO (Médecin Coordinateur du Centre d'Investigation Clinique)
Dr Alina CIUCHETE (Médecin attaché du Centre d'Investigation Clinique)

Coordonnées du lieu de recherche.
Centre d'Investigation Clinique St-Antoine – CIC-9304
184 rue du Fbg St-Antoine
75012 – Paris
Tel : 01 49 28 22 03, Fax : 01 49 28 21 08
Mél : contact.cic@sat.aphp.fr

Autres services impliqués dans le projet.

Unité INSERM partenaire de la recherche: François CAMBIEN (Directeur)
UMR S 525 Génétique Épidémiologique et Moléculaire des Pathologies Cardiovasculaires
Faculté de Médecine Pierre et Marie Curie – Pitié Salpêtrière
91 Boulevard de l'Hôpital. 75634 PARIS CEDEX 13
Tél : 01 40 77 97 25
Fax : 01 40 77 97 28
Mél : cambien@chups.jussieu.fr

Jean-Sébastien HULOT (Responsable projet)
U621 et Département de Pharmacologie. Faculté de Médecine Pierre et Marie Curie – Pitié Salpêtrière
91 Boulevard de l'Hôpital. 75634 PARIS CEDEX 13
Tél : 01 40 77 96 49
Fax : 01 40 77 96 45
Mél : jean-sebastien.hulot@psl.aphp.fr

Laboratoire de dosage

Dr Jean-Claude Alvarez
Laboratoire de Biochimie Pharmacologie et Toxicologie
Faculté de Médecine Paris-Ile-de-France-Ouest - Université de Versailles Saint-Quentin
CHU Raymond Poincaré, AP-HP, 104 Boulevard R. Poincaré, 92380 Garches
Tél : 01 47 10 79 38
Fax : 01 47 10 79 23
Mél : jean-claude.alvarez@rpc.aphp.fr

Responsable du monitoring sur site

Jennifer GALTIE
Centre d'Investigation Clinique St-Antoine – CIC-9304
184 rue du Fbg St-Antoine
75012 – Paris
Tel : 01 49 28 22 03, Fax : 01 49 28 21 08
Mél : secretariat-cic.sat@sat.aphp.fr

1. **Informations générales** ........ Error! Bookmark not defined.
1.1 Résumé ........ 5
1.2 Page de signature du protocole ........ 7
2. **Justification scientifique et description générale de la recherche** ........ 8
2.1 Dénomination et description du ou des médicaments expérimentaux. ........ 8
2.2 Résumé des résultats des essais non cliniques et des essais cliniques disponibles et pertinents au regard de la recherche biomédicale concernée. ........ 8
2.2.1 Médicaments allongeant la durée de l'intervalle QT : ........ 9
2.2.2 Torsades de pointes d'origine médicamenteuse : ........ 9
2.3 Résumé des bénéfices et des risques prévisibles et connus pour les personnes se prêtant à la recherche. ........ 11
2.4 Description et justification de la voie d'administration, de la posologie, du schéma d'administration et de la durée de traitement. ........ 11
2.5 Description de la population à étudier. ........ 11
2.6 Références à la littérature scientifique et aux données pertinentes servant de référence pour la recherche. ........ 11
2.7 Déclaration indiquant que la recherche sera conduite conformément au protocole, aux bonnes pratiques cliniques et aux dispositions législatives et réglementaires en vigueur. ........ 12
3. **Objectifs de la recherche** ........ 12
4. **Conception de la recherche** ........ 13
4.1 Enoncé précis des critères d'évaluation principaux et, le cas échéant, des critères d'évaluation secondaires. ........ 13
4.2 Description de la méthodologie de la recherche, accompagnée de sa présentation schématique précisant notamment les visites et les examens prévus. ........ 14
4.3 Description des mesures prises pour réduire et éviter les biais incluant notamment ........ 15
4.4 Description de la posologie et des modalités d'administration du ou des médicaments expérimentaux. Description de la forme unitaire, du conditionnement et de l'étiquetage du ou des médicaments expérimentaux. ........ 16
4.5 Durée prévue de participation des personnes et description de la chronologie et de la durée de toutes les périodes de l'essai, y compris le suivi, le cas échéant. ........ 16
4.6 Description des règles d'arrêt définitif ou temporaire ........ 16
4.7 Procédure d'arrêt prématuré de traitement correspondant à l'arrêt du traitement par le médicament expérimental, et procédure d'exclusion de la recherche correspondant à l'arrêt du traitement et du suivi de la personne dans le cadre de la recherche ........ 17
4.8 Procédures de comptabilité du ou des médicaments expérimentaux. ........ 17
4.9 Dispositions mises en oeuvre en vue du maintien de l'insu et procédures de levée de l'insu, le cas échéant. ........ 17
4.10 Identification de toutes les données à recueillir directement dans les cahiers d'observation, qui seront considérées comme des données source. ........ 17
5. **Sélection et exclusion des personnes de la recherche** ........ 18
5.1 Critères d'inclusion des personnes qui se prêtent à la recherche ........ 18
5.2 Critères de non- inclusion des personnes qui se prêtent à la recherche ........ 18
6. **Traitement administré aux personnes qui se prêtent à la recherche** ........ 18
6.1 Description du ou des traitements nécessaires à la réalisation de la recherche. ........ 18
6.2 Médicaments et traitements autorisés et interdits dans le cadre du protocole, y compris les médicaments de secours. ........ 18
6.3 Méthodes de suivi de l'observance au traitement. ........ 19
6.4 Conditions de stockage des médicaments expérimentaux. ........ 19
6.5 Conditions de dispensation des médicaments expérimentaux ........ 19
7. **Evaluation de l'efficacité** ........ 19
7.1 Description des paramètres d'évaluation de l'efficacité. ........ 19
7.2 Méthodes et calendrier prévus pour mesurer, recueillir et analyser les paramètres d'évaluation de l'efficacité. ........ 19
8. **Evaluation de la sécurité** ........ 19
8.1 Description des paramètres d'évaluation de la sécurité ........ 19
8.2 Méthodes et calendrier prévus pour mesurer, recueillir et analyser les paramètres d'évaluation de la sécurité ........ 20
8.2.1 Comité de pilotage ........ 20
8.2.2 Suivi des paramètres de sécurité ........ 20
8.3 Procédures mises en place en vue de l'enregistrement et de la notification des évènements indésirables ........ 20
8.3.1 Evènements indésirables non graves : ........ 20
8.3.2 Evènements indésirables graves (EIG) : cf annexe 4 ........ 20
8.4 Modalités et durée du suivi des personnes suite à la survenue d'évènements indésirables ........ 22
9. **Statistiques** ........ 22

9.1 Description des méthodes statistiques prévues, y compris du calendrier des analyses intermédiaires prévues. ............22
9.2 Nombre prévu de personnes à inclure dans la recherche, et nombre prévu de personnes dans chaque lieu de recherches avec sa justification statistique. ............23
9.3 Degré de signification statistique prévu. ............23
9.4 Critères statistiques d'arrêt de la recherche. ............23
9.5 Méthode de prise en compte des données manquantes, inutilisées ou non valides............23
9.6 Choix des personnes à inclure dans les analyses............23
**10. Droit d'accès aux données et documents source ............24**
**11. Contrôle et assurance de la qualité ............24**
11.1 Procédures de monitoring............24
11.2 Transcription des données dans le cahier d'observation ............24
**12. Considérations légales et éthiques............25**
12.1 Demande d'autorisation auprès de l'Afssaps ............25
12.2 Demande d'avis au Comité de Protection des Personnes............25
12.3 Modifications ............25
12.4 Déclaration CNIL ............26
12.5 Note d'information et Consentement éclairé............26
12.6 Rapport final de la recherche............26
**13. Traitement des données et conservation des documents et des données relatives à la recherche ............26**
**14. Assurance et engagement scientifique............27**
14.1 Assurance ............27
14.2 Engagement scientifique ............27
**15. Règles relatives à la publication ............27**
**16. Références bibliographiques.............28**
**17. Annexes.............30**
17.1 Annexe 1 : Carte patient............30
17.2 Annexe 2 : Genome-wide association studies (GWAS) ............30
17.3 Annexe 3 : Annonce de recrutement ............32
17.4 Annexe 4 : Formulaire de déclaration d'EIG ............33
17.5 Annexe 5 : Annexe pharmaceutique et fiche Vidal du Sotalol ............35
17.6 Annexe 6 : Prélèvements sanguins............39

# 1. INFORMATIONS GENERALES

## 1.1 Résumé

- Titre : identification des sources génétiques de variabilité de l'adaptation de la repolarisation ventriculaire a un stimulus pharmacologique et physiologique dans une population apparemment normale

- Recherche biomédicale n° P071007

- Nom et coordonnées de l'investigateur coordonnateur : Dr Beny CHARBIT.
Centre d'Investigation Clinique – Service de Pharmacologie
Hôpital St-Antoine
184 rue du Fbg St-Antoine - 75012 – Paris
Tél : 01 49 28 22 03 ; Fax : 01 49 28 21 08 ; Mél : beny.charbit@sat.aphp.fr

- Nom et coordonnées du responsable scientifique: Jean-Sébastien HULOT. U621 et Département de Pharmacologie. Hôpital de la Pitié Salpêtrière
91 Boulevard de l'Hôpital. 75634 PARIS CEDEX 13
Tél : 01 40 77 96 49, Fax : 01 40 77 96 45, Mél : jean-sebastien.hulot@psl.aphp.fr

- Justification :

L'allongement de l'intervalle QT de l'ECG, durant l'administration de médicaments, en particulier non cardiovasculaires, est un problème de santé publique compte tenu du nombre élevé de molécules concernées ainsi que de la gravité potentielle de cet évènement indésirable pouvant conduire à une mort subite par torsade de pointes. Malgré les progrès du diagnostic génétique des syndromes du QT long (SQTL) congénital, il n'est à ce jour le plus souvent pas possible de prédire ce type d'accident. En effet, on ne retrouve que rarement des mutations connues du SQTL congénital chez les sujets ayant développé ce type d'accident. Cependant, des variants génétiques, sur des gènes codant ou non pour des canaux ioniques influençant la repolarisation, ont été ponctuellement retrouvés chez ces sujets et il a aussi été montré que des variants génétiques étaient associés aux extrêmes de la distribution de l'intervalle QTc au repos dans la population générale. De nombreux arguments cliniques et expérimentaux indiquent que, dans les SQTL congénitaux, plus que la durée de QTc au repos, c'est la réponse au blocage pharmacologique des canaux repolarisants et l'adaptation de QTc à des variations de fréquence cardiaque, notamment sous l'effet de la stimulation sympathique, qui constituent le primum movens de l'arythmogénèse. Ces facteurs pharmacologiques et dynamiques pourraient jouer un rôle dans l'identification des sujets à risque de torsades de pointes d'origine médicamenteuse dans la population générale.
La stratégie génétique envisagée pour la présente étude se fonde sur le fait que les gènes potentiellement impliqués dans les variations de l'intervalle QT (gènes candidats) sont seulement partiellement connus. L'approche doit donc permettre de découvrir de nouveaux gènes. Elle consistera en une étude d'association pangénomique (Genome-Wide Association Study – GWAS) utilisant une puce de génotypage comportant plus de 300.000 marqueurs (Illumina) qui devrait permettre d'identifier des gènes dont l'implication dans les phénotype étudiés est insoupçonnée.

- Objectifs de la recherche :

Appliquer une approche pangénomique dans la recherche de facteurs génétiques (gènes et variants) impliqués dans les modifications de l'intervalle QT/QTc (réponses extrêmes et continuum des réponses) en réponse à une stimulation pharmacologique (Sotalol) et

physiologique (stimulation sympathique) dans la population générale apparemment bien portante.

- Critères de sélection

Sujets européens et nord-africains âgés de 18 à 60 ans, apparemment sains, indemnes de toute pathologie significative et de tout traitement au long cours.

- Nombre de sujets nécessaire : 1000 participants

- Durée de la recherche : 3 ans,

- Durée de participation de chaque volontaire : environ 6 heures

- Méthodologie

Étude transversale de 1000 sujets apparemment sains qui recevront une dose unique de Sotalol et auront une épreuve d'effort sur bicyclette ergonomique, une stimulation auditive et un prélèvement d'ADN. Le génotypage sera réalisé par une approche pangénomique sur plateforme Illumina.

- Examens requis spécifiquement pour la recherche

Prélèvement de 30 ml de sang, épreuves d'effort et enregistrements d'électrocardiogrammes.

- Critère d'évaluation principal et critères secondaires

Le critère principal est l'allongement de QTc 3 heures après une prise orale de 80 mg de Sotalol (pic attendu de l'effet).
Les critères secondaires sont l'adaptation de QTc à l'épreuve d'effort et à une stimulation auditive.

<u>Résultats attendus et implications potentielles.</u>

L'identification de profils phénotypiques et génotypiques associés à une réponse au Sotalol excessive ou sortant de la norme, pourrait permettre de disposer de tests diagnostics visant, à terme, à dépister les sujets à risque et prévenir les torsades de pointes médicamenteuses.

## 1.2 Page de signature du protocole

Page de SIGNATURE D'UN PROTOCOLE de recherche biomédicale
par l'investigateur coordonnateur et le représentant du promoteur

Recherche biomédicale n°P071001

Titre : « Identification des sources génétiques de variabilité de l'adaptation de la repolarisation ventriculaire à un stimulus pharmacologique et physiologique dans une population apparemment normale »

Version N° 1 du : 16 octobre 2007

L'investigateur principal :

Dr Beny CHARBIT
Centre d'Investigation Clinique – Service de Pharmacologie
Hôpital St-Antoine
184 rue du Fbg St-Antoine - 75012 – Paris
Tél : 01 49 28 22 03 ; Fax : 01 49 28 21 08 ; Mél : beny.charbit@sat.aphp.fr

L'investigateur coordonnateur autorise également la diffusion de son identité et de ses coordonnées dans le répertoire des recherches biomédicales

Date : ……………/………/………..

Signature :

Le promoteur :

Dr Olivier CHASSANY
Assistance publique – hôpitaux de Paris
Délégation Interrégionale à la Recherche Clinique Date : ……………/………/………..
Hôpital Saint Louis
Signature :

# 2. JUSTIFICATION SCIENTIFIQUE ET DESCRIPTION GENERALE DE LA RECHERCHE

## 2.1 Dénomination et description du ou des médicaments expérimentaux.

Le Sotalol est un béta-bloquant antiarythmique de classe III indiqué principalement dans le traitement des tachycardies supraventriculaires paroxystiques. Le Sotalol a été choisi car ses effets sur l'allongement de QTc sont bien connus et que ce médicament a une faible variabilité pharmacocinétique (absorption complète, biodisponibilité voisine de 100 %, pas de métabolisation, peu liée aux protéines plasmatiques).[1] Cette molécule a été largement utilisée au CIC St-Antoine dans des études humaines.[1-4]
Le seul médicament administré lors de cette étude sera du Sotalol à la dose de 80mg en prise unique par voie orale.

## 2.2 Résumé des résultats des essais non cliniques et des essais cliniques disponibles et pertinents au regard de la recherche biomédicale concernée.

*Généralités*
L'allongement de l'intervalle QT sur l'électrocardiogramme (ECG) reflète l'augmentation de la durée de la repolarisation ventriculaire. Celle-ci résulte de modifications de courants ioniques au travers de la membrane des cardiomyocytes. L'allongement excessif de l'intervalle QT est un facteur d'inhomogénéité de la repolarisation ventriculaire, elle-même source d'arythmogénèse.[5] Celle-ci survient sous la forme d'un trouble du rythme spécifique dénommé torsade de pointes qui peut être spontanément résolutif mais est potentiellement mortel par sa capacité à dégénérer en fibrillation ventriculaire.[6]
Le syndrome du QT long existe sous deux entités : le syndrome du QT long (SQTL) congénital, qui regroupe plusieurs pathologies liées à des mutations sur des canaux ioniques cardiaques appelées aussi canalopathies, et le QT long acquis, qui est lié soit à une maladie générale (hypertrophie ventriculaire, accident vasculaire cérébral ; ou non cardio-vasculaire : diabète, cirrhose…) soit à une prise de médicaments interférant avec la repolarisation ventriculaire, le plus souvent en bloquant la conduction de ou plusieurs canaux potassiques.

*Syndrome du QT long congénital : maladie génétique*
Le syndrome du QT long congénital (SQTL congénital) regroupe un ensemble de cardiopathies congénitales arythmogènes dont la prévalence est inférieure à 1/1 000.[7,8] Des polymorphismes génétiques (voir aussi http://www.qtsyndrome.ch/bio.html) ont été identifiés dans des familles de SQTL congénital,[9-11] dont le type influence le pronostic.[12] Par ailleurs, plusieurs études ont mis en évidence des relations avec certains variants de gènes codant pour des canaux ioniques cardiaques et la durée de l'intervalle QT.[13,14]
Pfeufer et al.[14] ont identifié plusieurs variants à partir d'une base de données initiale de 6640 sujets de laquelle une première population de 689 sujets a été étudiée puis 3277 sujets ont participé à l'échantillon de validation. Cette étude a retrouvé des relations entre des variants des gènes KCNQ1, KCNH2, KCNE1 qui allongent ou au contraire raccourcissent la durée de QT. Ces variants étaient associés à des variations de QT de 2 à 4 ms respectivement à l'état hétérozygote ou homozygote. L'équipe de l'unité U 582 à la Pitié-Salpêtrière a récemment retrouvé des différences de répartition de variants alléliques de KCNQ1, KCNE1, KCNH2 et SCN5A entre deux sous populations de 200 sujets chacune sélectionnées à partir d'une

population de 2008 sujets sains comme ayant des valeurs de QT les plus courtes ou les plus longues.[13]
Récemment, de nouvelles approches pangénomique ont permis d'identifier d'autres sources de variabilité de l'intervalle QT. En particulier, une relation entre la durée de la repolarisation et un variant génétique fréquent de *NOS1AP (CAPON)*, un régulateur de la NOsynthase neuronale, a été récemment retrouvée.[15]
La repolarisation ventriculaire, et par extension certaines arythmies cardiaques, sont donc en partie sous l'influence de facteurs génétiques multiples.[16]

*Syndrome du QT long acquis.*

2.2.1 Médicaments allongeant la durée de l'intervalle QT :
De très nombreux médicaments allongent la durée de la repolarisation ventriculaire (voir une liste sur http://www.torsades.org/medical-pros/drug-lists/drug-lists.htm). Certains de ces médicaments sont des antiarythmiques qui étaient initialement destinés à allonger, à des fins thérapeutiques, la durée des périodes réfractaires par le biais d'un allongement de la repolarisation mais ils se sont révélés être également arythmogènes[17] ce qui en limite l'intérêt thérapeutique. Surtout, des médicaments non-cardiovasculaires parmi lesquels, certains anti-infectieux, des neuroleptiques ou des antihistaminiques[18] ont révélé un potentiel arythmogène rare mais dont la potentielle dangerosité justifie aujourd'hui l'évaluation systématique de l'effet de tout nouveau médicament sur la durée de la repolarisation ventriculaire.[19,20] La majorité de ces médicaments qui allongent la durée de QT chez l'homme induisent une inhibition des courants potassiques IKs ou IKr *in vitro*. Il existe cependant une très grande variabilité de l'allongement de l'intervalle QT sous l'effet de ces médicaments *in vivo* chez l'homme. De telle sorte qu'il n'existe aujourd'hui pas de moyen fiable de prévoir qui est exposé au risque de torsades de pointes médicamenteuses.

2.2.2 Torsades de pointes d'origine médicamenteuse :
Les patients victimes de ce type d'accident médicamenteux sont le plus souvent initialement bien portants et ne révèlent un trouble du rythme cardiaque qu'à l'occasion d'une exposition à un médicament, notamment non cardiovasculaire, bloqueur des canaux potassiques. On peut citer l'exemple de la terfenadine, médicament antihistaminique depuis retiré du marché et qui a entraîné le décès de plusieurs sujets par ailleurs en bonne santé.[21] Ce médicament, bloqueur des canaux IKr provoquait un allongement du QT non détecté lors du développement de la molécule. Aujourd'hui, la problématique clinique est celle visant à identifier, avant l'administration d'un médicament potentiellement toxique, des facteurs de susceptibilité individuelle à ce type de trouble du rythme.

*2.2.2.1 Concept de réserve de repolarisation :*

L'administration d'un médicament bloquant faiblement les canaux potassiques produit chez certains sujets susceptibles un allongement de la repolarisation important du fait d'une faible « réserve de repolarisation ». Du fait de cette faible réserve de repolarisation, ces sujets, phénotypiquement normaux à l'état de base, ont des réponses excessives lors de l'administration de bloqueurs de canaux potassiques et seulement dans ces conditions. Ces sujets peuvent ainsi révéler leur appartenance à des familles de SQTL congénitaux non encore identifiés ou être atteints de mutations ponctuelles.
Il a été suggéré que cette réserve de repolarisation pouvait se traduire cliniquement par la mesure de l'intervalle QT au repos. Ainsi un patient ayant un QT allongé avant de recevoir un médicament allongeant le QT sera plus à risque de développer une torsade de pointes.[18] Mais cette notion physiopathologique, parfois vérifiée pour les

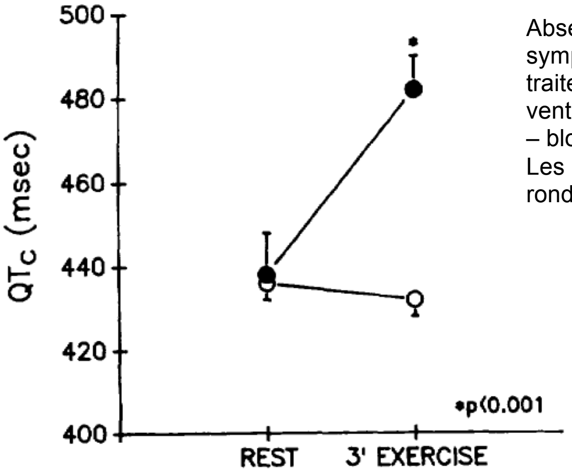


Absence d'adaptation de l'intervalle QTc à une stimulation sympathique par un exercice chez des sujets hors traitement médicamenteux mais ayant fait une tachycardie ventriculaire polymorphe sous antiarythmique de classe Ia – bloqueurs de canaux potassiques (d'après la référence 25). Les patients porteurs d'un SQTL sont représentés par les ronds pleins.

SQTL congénitaux n'est en règle pas retrouvée dans la population générale. Si il arrive que la survenue de Torsades de Pointes médicamenteuse révèle un SQTL congénital « silencieux » à l'état basal,[22] les torsade de pointes restent souvent un accident médicamenteux imprévisible hors de ce contexte très particulier et notre objectif est d'évaluer si des facteurs génétiques modulent non pas la durée de l'intervalle QT à l'état basal mais son adaptation dynamique lors de tests de provocation physiologique et pharmacologiques.

*2.2.2.2 Adaptation du QT à l'effort : une évaluation de la réserve de repolarisation ?*

Les anomalies de morphologie de l'onde T et de la durée de QT à l'effort ou lors d'une stimulation sympathique sont des facteurs du risque arythmogène des SQTL congénitaux [23,24]et, d'une manière beaucoup plus intéressante dans notre perspective, l'inadaptation de la repolarisation à un changement rapide de fréquence cardiaque lors d'un exercice physique est un marqueur du risque de torsades de pointes médicamenteuses (figure ci-contre).[25] Récemment, l'évolution du QT après des efforts calibrés a été retrouvée comme différente entre des patients porteurs d'un syndrome du QT long de type1 par comparaison à des volontaires sains.[26] Plus précisément, pendant une manœuvre calibré de handgrip durant 3 minutes, le QT se raccourci mais à la fin de la manœuvre, les auteurs observent un allongement exagéré du QT « QT overshoot » chez les patients atteints d'un SQTL congénital de type 1 par comparaison à des sujets sains. Ce mécanisme pourrait expliquer la survenue d'arythmie en rapport avec l'effort physique chez ces patients.[26] Une autre étude a retrouvé également un allongement excessif de l'intervalle QT après la fin d'un exercice physique chez les patients LQT1 et LQT2 par rapport à des sujets sains.[27] Dans ce même travail, tous les patients porteurs de mutations canalaires avaient à l'effort un QT plus long que les sujets contrôles. Les auteurs concluant que la mesure du QT à l'effort pouvait améliorer la détection des patients porteurs d'un SQTL congénital.[27] De manière plus générale, l'adaptation du cœur à l'effort est liée au risque de mort subite, elle-même souvent causée par un épisode arythmique.[28]

A ce jour, aucune étude publiée n'a exploré les relations entre ce marqueur dynamique du risque arythmogène et des polymorphismes génétiques communs ou des mutations ponctuelles.

Au total, l'originalité de l'approche phénotypique développée ici est de sensibiliser l'identification d'un phénotype anormal sur la dynamique de la repolarisation (sous l'effet du Sotalol, d'une part, et d'une stimulation sympathique, d'autre part) et, contrairement à la seule mesure de la durée de l'intervalle QT au repos, de viser l'identification de phénotypes « forts ». C'est bien la population générale apparemment bien portante qui est concernée et non la population des SQTL congénitaux connus puisque cette population apparemment normale est elle aussi exposée aux risques de torsades de pointes médicamenteuses.

## 2.3 Résumé des bénéfices et des risques prévisibles et connus pour les personnes se prêtant à la recherche.

Les risques associés à la prise d'une dose unique de 80 mg de Sotalol sont minimaux et il faut rappeler que la posologie initiale recommandée de Sotalol est de 80 mg 2 fois par jour, la plupart des patients répondant à une posologie comprise entre 160 et 320 mg en 2 prises par jour. Le Sotalol est couramment prescrit en ville sans surveillance particulière initiale dans la prévention des récidives de tachycardies supra-ventriculaires. Nous administrerons par ailleurs une dose faible et unique.
Cette molécule a été largement utilisée au CIC St-Antoine dans des études humaines.[1-4] Aucun évènement indésirable rythmique n'est jamais survenu au cours de ces études même si 2 volontaires présumées saines ont eu une réponse excessive, restée asymptomatique.[2]

Malgré cela, nous assurerons un monitoring permanent de l'ECG durant l'étude et avons choisi un critère de sortie du CIC fondé sur le retour de l'intervalle QTc dans des limites acceptables. Enfin, nous disposons au CIC St-Antoine d'une procédure de gestion des torsades de pointes et d'un contact permanent avec l'Unité de Soins Intensifs Cardiologiques située 3 étages au dessus dans le même bâtiment.
Les volontaires participant à l'étude seront indemnisés de 140 € pour les contraintes imposées pendant l'étude. Le montant de l'indemnisation sera communiqué au CPP et sera déclaré dans le fichier national géré par l'Agence Française de Sécurité Sanitaire des Produits de Santé.

## 2.4 Description et justification de la voie d'administration, de la posologie, du schéma d'administration et de la durée de traitement.

La voie orale a été retenue dans cette étude pour sa simplicité et parce qu'elle est la voie d'administration classique de ce médicament dont il existe une forme injectable. La posologie de 80 mg est justifiée car elle est la quantité classiquement prescrite à l'initiation du traitement. A cette posologie, il existe un allongement de l'intervalle QT qui nous permettra de répondre à notre objectif principal. Nous avons également retenu la dose unique pour des raisons de simplicité et de sécurité.

## 2.5 Description de la population à étudier.

Les sujets seront recrutés au sein de la base de données des volontaires du CIC St-Antoine, par voie d'affichage et communication orale locale et par petites annonces (annexe 3) dans un ou 2 journaux gratuits en cas de difficulté de recrutement. L'équipe du CIC a récemment réalisé sous la conduite du Dr Beny Charbit une étude ayant permis en 9 mois l'inclusion de 1100 sujets séropositifs VIH (en cours de soumission pour publication) pour un simple ECG de repos.
Il est prévu de n'inclure que des sujets dont l'origine géographique est soit européenne soit nord-Africaine ceci afin de limiter l'hétérogénéité génétique. Au sens origine géographique nous entendons que les parents du sujet doivent avoir la même origine.

## 2.6 Références à la littérature scientifique et aux données pertinentes servant de référence pour la recherche.

Ces références ont été développées dans les paragraphes précédents.

## 2.7 Déclaration indiquant que la recherche sera conduite conformément au protocole, aux bonnes pratiques cliniques et aux dispositions législatives et réglementaires en vigueur.

Cette étude entre dans le cadre d'une « recherche biomédicale au sens de l'article L.1121-1 du Code de la Santé Publique. Le Promoteur de cette étude sera l'Assistance Publique – Hôpitaux de Paris.
L'investigateur expliquera soigneusement aux sujets le but et les modalités de participation à l'étude. Il leur remettra le formulaire d'information et de consentement et devra obtenir leur consentement écrit avant de les faire entrer dans l'essai. Les sujets seront informés du fait qu'ils peuvent se retirer de l'essai à tout moment sans avoir à se justifier.
Le promoteur de l'étude soumettra au Comité de Protection des Personnes (CPP) de Saint-Antoine et à l'agence française de sécurité sanitaire des produits de santé (AFSSAPS), le dossier constitué des documents requis par celui-ci et la réglementation. Un avis favorable devra être obtenu auprès de ce CPP et de l'agence de sécurité sanitaire des produits de santé afin de pouvoir réaliser l'étude.
Une fois l'étude débutée, le CPP et l'AFSSAPS devront être tenu informés de tout événement susceptible d'affecter la sécurité des sujets.
L'anonymat des sujets devra être respecté conformément à la loi ; le recueil et le traitement des données se feront dans ce cadre.
Toute personne ayant accès aux consentements et aux documents source est tenue au secret professionnel.

L'U525 est un centre de référence d'épidémiologie génétique et dispose de toutes les procédures et autorisations *ad hoc* pour la protection des personnes en matière de prélèvements génétiques.

# 3. OBJECTIFS DE LA RECHERCHE

L'objectif principal est d'appliquer une approche de génotypage pangénomique dans la recherche de facteurs génétiques (gènes et variants) impliqués dans les modifications extrêmes de l'intervalle QT de l'électrocardiogramme en réponse à une stimulation pharmacologique (Sotalol) et physiologique (stimulation sympathique) dans la population générale apparemment bien portante. Une caractérisation phénotypique originale fondée, sur la dynamique de la repolarisation ventriculaire (et non sur la durée de QT au repos) sera utilisée en visant à terme des facteurs génétiques prédictifs de syndrome du QT long <u>acquis</u>. Il est prévu de recruter au total 1000 sujets.
L'objectif secondaire est de constituer une base de ressources biologique, d'une part, et phénotypique, d'autre part, qui permettront de définir des phénotypes de réponse aux tests dynamiques mis en œuvre et de rechercher des associations entre ces phénotypes de réponse éléctrocardiographiques et des mutations ou des polymorphismes d'intérêt.

<u>Retombées en terme de santé dans la prise en charge du patient.</u>
Cette étude est susceptible d'identifier des variants génétiques prédictifs d'une réponse excessive à l'administration d'un médicament allongeant la durée de la repolarisation ventriculaire par le mécanisme principalement mis en cause dans les syndromes du QT long acquis, le blocage de Ikr.
Les tests phénotypiques entrepris ne l'ont jamais été à une grande échelle et l'étude permettra donc, au minimum, de déterminer la fréquence des réponses excessives dans la population générale apparemment bien portante (et non plus seulement chez des gens sélectionnés par leur réponse proarythmique aux bloqueurs d'Ikr).

Ce projet est donc susceptible d'apporter une innovation – éventuellement valorisable – dans le diagnostic de susceptibilité des torsades de pointes médicamenteuses. Il faut souligner le fait que les patients exposés à ce risque sont ceux de la population générale saine ou relativement saine qui se voit prescrire des médicaments pour des affections souvent passagères survenant sur un état physiologique normal : antihistaminiques dans les allergies communes, antibiotiques dans les sinusites et bronchites communes, antidépresseurs dans la dépression notamment. À ce titre, la réalisation de l'étude dans une population apparemment saine est pleinement justifiée.
Enfin, la bio-ressource et la base de données ECG constituées au cours de ce projet pourra être exploitée bien au delà de l'étude initiale décrite ici. Dans cette perspective, un Comité Scientifique sera constitué à moyen terme, regroupant les 4 responsables du projet actuel pour déterminer les règles d'utilisation de ces bases pour des études ultérieures de génétique prédictive.

# 4. CONCEPTION DE LA RECHERCHE

## 4.1 Enoncé précis des critères d'évaluation principaux et, le cas échéant, des critères d'évaluation secondaires.

*Partie clinique*
Le critère de jugement principal sera l'allongement de la durée de l'intervalle QT corrigé (QTcf) 3 h (pic présumé de l'effet du Sotalol[1,29]) après la prise orale unique d'une faible dose de 80 mg de Sotalol. Une analyse de sensibilité sera effectuée en utilisant la méthode de correction populationnelle proposée par Malik.[30] Une période témoin placebo chez le même sujet n'est pas nécessaire compte tenu des données antérieures sur les effets du Sotalol[1,29].
Les électrocardiogrammes seront effectués avec une machine de marque Cardionics système d'enregistrement digital du signal, relié à un micro-ordinateur. Les enregistrements seront effectués sur des sujets au repos, allongés, sur 12 dérivations. Les éléments de l'ECG (intervalles RR & QT) sont mesurés automatiquement à l'aide de curseurs. Cette mesure automatique est débrayable et la position des curseurs peut être modifiée par l'opérateur chargé de la validation. L'analyse des couples RR / QT sera réalisée après la fin de l'étude. La lecture sera faite pour chaque sujet par le même opérateur, en aveugle et sur la même dérivation (correspondant à celle où l'onde T est la plus lisible et a la plus grande amplitude, en général V2, V3 ou V4). Le couple RR / QT sera le résultat de la moyenne des intervalles QT et des intervalles RR correspondants (RR précédant le QT mesuré) mesurés pendant une période de 30 secondes.
Afin d'obtenir une mesure de l'adaptation de l'intervalle QTc lors d'une stimulation sympathique, nous réaliserons une épreuve d'effort sur bicyclette ergonomique qui permet d'augmenter transitoirement la fréquence cardiaque de repos. L'épreuve d'effort se déroulera de la façon suivante. Le sujet sera installé sur la bicyclette et l'enregistrement ECG sera débuté. On demandera au sujet de commencer à pédaler (charge à 60 watts). Puis la charge sera augmentée de 15 watts toutes les 2 minutes sous contrôle de la fréquence cardiaque. L'épreuve sera arrêtée lorsque la fréquence cardiaque aura dépassé 110 battements/min. L'évolution du QT à la fin de cette manœuvre et au cours des premières minutes après la fin de cette manœuvre nous permettra d'évaluer l'adaptation du QT au cours et au décours d'une stimulation sympathique (critère secondaire). Un test similaire sera effectué par un stimulus auditif. Il consistera à proposer aux participants d'écouter à l'aide d'un casque, un morceau de musique calme en leur demandant de fermer les yeux et de tenter de se détendre. Au bout d'environ 3-4 minutes, sans que les sujets aient été prévenus, nous remplacerons la musique par un bruit fort et désagréable (sonnerie de réveil). Ce type de stimulation auditive a été choisi car il représente chez certains patients porteurs d'un SQTL (en particulier de type 2) un mode possible de déclenchement d'épisode arythmique.[31]

Ces investigations permettront de disposer pour chaque sujet de plusieurs critères de caractérisation phénotypiques :
ΔQTcf 3 h après la prise de 80 mg de Sotalol (critère principal)
ΔQTcf au maximum et au décours d'une épreuve d'effort
ΔQTcf au maximum de variation du test de stimulation auditif

*Partie génétique :*
Le coût des puces de génotypage pangénomique demeure important, environ 250 euros/puce et il n'est pas envisageable, du moins dans un premier temps, de génotyper les 1000 individus participant à l'étude. Pour contourner cette difficulté nous envisageons de pooler les ADN en fonction de la durée de l'intervalle QTcf sous Sotalol et du sexe. Et de quantifier la fréquence des allèles sur les ADN poolés (annexe 2). Grâce à une subvention de l'ANR, nous avons mis au point l'approche de GWAS sur pools d'ADN et l'appliquons à diverses pathologies du système cardiovasculaire. (www.agence-nationale-recherche.fr/documents/aap/2006/selection/blanc.pdf).
La perte de puissance liée à l'analyse des ADN poolés comparativement à l'analyse individuelle est modeste si la technique de pooling est correcte.
(http://www.ecgene.net/files/POOLCA_FINAL_partB.pdf).

## 4.2 Description de la méthodologie de la recherche, accompagnée de sa présentation schématique précisant notamment les visites et les examens prévus.

Il s'agit d'une étude transversale en vue de la constitution d'une bio-ressource génétique, d'une part, et d'une base de données phénotypiques, d'autre part, permettant de faire des études d'association entre sources de variations phénotypiques (modifications de l'intervalle QT de l'électrocardiogramme en réponse à une stimulation) et variants génétiques (en utilisant une approche pangénomique).
Chaque sujet sera étudié au cours d'un seul passage au CIC St-Antoine (autorisation de lieu pour recherches biomédicales n° 10183 S et M) dénommé Visite 1.
Les volontaires seront convoqués au CIC, à 8 heures à jeun, pour une durée de 6 à 7 heures permettant successivement :
D'obtenir le consentement éclairé et écrit du sujet.
De réaliser un questionnaire médical relevant notamment des informations anthropologiques (âge, BMI, sexe, origine géographique,[32]…), sur les habitudes de vie (tabagisme, prise médicamenteuse…) et un examen médical incluant en particulier une mesure de la pression artérielle et de la fréquence cardiaque.
Un test urinaire de grossesse sera réalisé chez les femmes en âge de procréer.
La date des dernières règles sera relevée.
De réaliser un électrocardiogramme digital avec procédure de diagnostic automatisé immédiate validée par un médecin.
En l'absence d'anomalie électrocardiographique significative (bloc de branche complet ou incomplet, bloc de conduction), et d'autres critères de non-inclusion, le volontaire sera définitivement inclus.
Un cathéter permettra d'effectuer un dosage de kaliémie et un prélèvement d'ADN.
L'enregistrement de l'électrocardiogramme 12-dérivations digital continu sera alors débuté.
Au cours de la première demi-heure d'enregistrement, deux tests d'adaptabilité du QT seront réalisés (cf. chapitre précédent):

- un effort modéré et calibré sur bicyclette ergonomique
- stimulus auditif calibré

A la fin de cette demi-heure, les volontaires recevront par voie orale 80 mg de Sotalol (Sotalol ; http://www.biam2.org/www/Sub1486.html)
Pendant toute la durée de l'hospitalisation l'électrocardiogramme sera surveillé par monitoring continu) et l'ECG 12 dérivations enregistré (Système Cardionics avec signal digital 500 Hz enregistré sur mémoire solide).
La pression artérielle sera surveillée toute les heures jusqu'à la sortie.
Trois heures après la prise du Sotalol, il sera réalisé un prélèvement sanguin pour dosage plasmatique du Sotalol. Un électrocardiogramme 12 dérivations sera imprimé à ce moment pour mesure du QT « pic ».
A T+3h, l'épreuve d'effort sur bicyclette ergonomique sera répétée puis un repas sera servi aux volontaires.

5 heures après la prise médicamenteuse, une mesure de l'intervalle QT sera réalisée. Si la valeur du QTcf est inférieure à sa valeur basale + 40msec, le volontaire sera autorisé à quitter le CIC. À défaut, un ECG sera à nouveau enregistré toutes les heures jusqu'à atteindre ce critère.

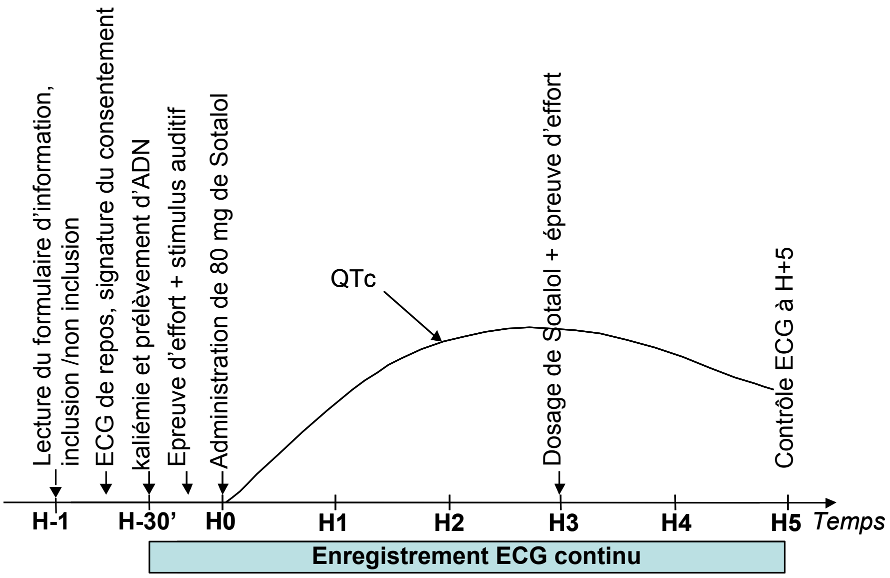


## 4.3 Description des mesures prises pour réduire et éviter les biais incluant notamment

a) Le tirage au sort
Non applicable.

b) Les méthodes de mise en insu.
Non applicable.

Les données cliniques et génétiques seront analysées séparément au CIC et à l'U 525 et en insu de leurs résultats respectifs de manière à limiter les biais statistiques.

## 4.4 Description de la posologie et des modalités d'administration du ou des médicaments expérimentaux. Description de la forme unitaire, du conditionnement et de l'étiquetage du ou des médicaments expérimentaux.

Une dose unique de Sotalol 80mg (Sotalol) sera administrée avec un verre d'eau aux volontaires par un(e) infirmièr(e) ou par un médecin du CIC.
Des enveloppes contenant un comprimé de Sotalol 80 mg pour une prise destinée à un sujet seront préparées à l'avance par la pharmacie.
L'étiquetage des enveloppes comportera le nom de l'étude, le lieu de la réalisation des lots, le n° patient, le N° de lot, la date de péremption + mentions Art R5123 CSP

Annexe pharmaceutique (annexe 5)

## 4.5 Durée prévue de participation des personnes et description de la chronologie et de la durée de toutes les périodes de l'essai, y compris le suivi, le cas échéant.

Suivi des patients :

| | Visite 1 |
|---|---|
| Lecture du formulaire d'information | X |
| Vérification des critères d'inclusion et de non-inclusion | X |
| ECG de repos | X |
| Signature du formulaire d'information et de consentement | X |
| Test urinaire de grossesse | X |
| Pose d'un cathéter, kaliémie, prélèvement pour génotypage | X |
| Début d'enregistrement d'ECG en continu | X |
| Epreuve d'effort, Stimulus auditif | X |
| Administration de 80 mg de Sotalol | X |
| Prélèvement sanguin à T3h (dosage plasmatique de Sotalol), épreuve d'effort | X |
| ECG de contrôle à T5h, autorisation de sortie du sujet | X |
| ECG de contrôle à T6h si nécessaire | X |

Prélèvements sanguins (annexe 6)

Durée totale prévisionnelle de la recherche (durée d'inclusion et durée de participation)
La durée de participation d'un volontaire sera d'une journée d'environ 6 à 7 heures.
L'étude est prévue pour durer 3 ans.

## 4.6 Description des règles d'arrêt définitif ou temporaire

- de la participation d'une personne à la recherche
Etant donné la particularité de cette étude qui ne dure que quelques heures pour chaque participant, l'arrêt de la participation n'interviendra qu'en cas de survenue d'un événement indésirable grave qui justifiera une prise en charge médicale adaptée et qui entraînera l'arrêt des prélèvements sanguins et tests d'adaptation.

- d'une partie ou de la totalité de la recherche

La recherche sera interrompue dans le cas de la survenue d'événements indésirables grave en particulier si 1 participant décède ou présente une complication sévère entraînant des conséquences durables à la suite de l'administration médicamenteuse. La recherche sera également interrompue dans le cas où plus de 2% des participants présenteraient un/des épisodes de torsades de Pointe.

## 4.7 Procédure d'arrêt prématuré de traitement correspondant à l'arrêt du traitement par le médicament expérimental, et procédure d'exclusion de la recherche correspondant à l'arrêt du traitement et du suivi de la personne dans le cadre de la recherche

a) Critères et modalités d'arrêt prématuré du traitement ou d'exclusion d'une personne de la recherche

Etant donné la particularité de cette étude qui ne dure que quelques heures pour chaque participant, l'arrêt de la participation n'interviendra qu'en cas de survenue d'un événement indésirable grave qui justifiera une prise en charge médicale adaptée et qui entraînera l'arrêt des prélèvements sanguins et tests d'adaptation.

b) Règles de levée d'insu le cas échéant

Non applicable

c) Modalités et calendrier de recueil pour ces données

Non applicable

d) Modalités de remplacement de ces personnes, le cas échéant

Pas de remplacement prévu.

e) Modalités de suivi de ces personnes.

Surveillance médicale par le médecin du CIC en relation avec les services spécialisés.

## 4.8 Procédures de comptabilité du ou des médicaments expérimentaux.

La comptabilité du traitement de l'étude sera effectuée par le personnel du CIC.

A l'issue de l'étude, les enveloppes de traitement et de secours (vides ou pleines) seront retournées à la pharmacie

## 4.9 Dispositions mises en oeuvre en vue du maintien de l'insu et procédures de levée de l'insu, le cas échéant.

Non applicable

## 4.10 Identification de toutes les données à recueillir directement dans les cahiers d'observation, qui seront considérées comme des données source.

Seront recueillies dans les cahiers d'observations les critères d'inclusion/non-inclusion, les informations démographiques, les heures de réalisation des tests et des prélèvements sanguins et d'administration du traitement.

# 5. SELECTION ET EXCLUSION DES PERSONNES DE LA RECHERCHE

## 5.1 Critères d'inclusion des personnes qui se prêtent à la recherche

- 50% d'hommes, 50% de femmes
- Âgés de 18 à 60 ans
- Origine européenne ou nord-africaine
- Indice de masse corporelle entre 19 et 29 kg/m²
- Obtention du consentement éclairé et écrit

## 5.2 Critères de non- inclusion des personnes qui se prêtent à la recherche

- Asthme
- Fréquence cardiaque < 50 bpm
- Hypotension artérielle avec pression artérielle systolique < 100 mm Hg.
- Bloc auriculo-ventriculaire (intervalle PR > 200 ms)
- Pathologie évolutive connue (HTA, insuffisance rénale, cardiaque, atteinte artérielle, maladie hépatique …)
- Phénomène de Raynaud
- Prise de médicament connus pour allonger la durée de QT (http://www.torsades.org/medical-pros/drug-lists/drug-lists.htm)
- Tous les traitements chroniques seront interdits sauf : contraception orale, antalgiques, vitamines et médicaments de confort n'allongeant pas le QT.
- Antécédent familial ou personnel connu de syndrome du QT long congénital ou de syncope inexpliquée
- QTc Fridericia (QTcf) > 450 ms. Le choix de la formule de correction de Fridericia repose sur le fait qu'elle minimise les erreurs liées à l'utilisation de la formule de Bazett.[33]
- Intervalle QT non mesurable sur l'ECG de repos ou bloc de branche (y-compris les blocs incomplets droits), QRS > 100 ms
- Période d'exclusion d'une autre recherche biomédicale (vérification du fichier national)
- Non affiliation à un régime de sécurité sociale (bénéficiaire ou ayant droit)
- Test urinaire de grossesse positif

# 6. TRAITEMENT ADMINISTRE AUX PERSONNES QUI SE PRETENT A LA RECHERCHE

## 6.1 Description du ou des traitements nécessaires à la réalisation de la recherche.

Une dose unique de Sotalol comprimé dosé à 80 mg sera administrée aux volontaires.

## 6.2 Médicaments et traitements autorisés et interdits dans le cadre du protocole, y compris les médicaments de secours.

Tous les traitements chroniques seront interdits sauf : contraception orale, antalgiques, vitamines et médicaments de confort n'allongeant pas le QT.
Prise de médicament connus pour allonger la durée de QT (http://www.torsades.org/medical-pros/drug-lists/drug-lists.htm).

## 6.3 Méthodes de suivi de l'observance au traitement.

Le traitement sera administré par un(e) infirmièr(e) ou par un médecin du CIC.

## 6.4 Conditions de stockage des médicaments expérimentaux.

Les enveloppes de traitement seront conservées à température ambiante dans la pharmacie du CIC.

## 6.5 Conditions de dispensation des médicaments expérimentaux

La pharmacie de l'hôpital Saint-Antoine délivrera au cic sur ordonnance libre mentionnant le nom de l'étude les enveloppes de traitement et de secours.
Il sera remis aux volontaires une « carte patient » (annexe 1)

# 7. EVALUATION DE L'EFFICACITE

## 7.1 Description des paramètres d'évaluation de l'efficacité

Non applicable en l'absence de mesure d'efficacité dans cette étude.

## 7.2 Méthodes et calendrier prévus pour mesurer, recueillir et analyser les paramètres d'évaluation de l'efficacité.

Non applicable en l'absence de mesure d'efficacité dans cette étude.

# 8. EVALUATION DE LA SECURITE

## 8.1 Description des paramètres d'évaluation de la sécurité

- Evènement indésirable

Toute manifestation nocive survenant chez une personne qui se prête à une recherche biomédicale que cette manifestation soit liée ou non à la recherche ou au produit sur lequel porte cette recherche.

- Effet indésirable d'un médicament expérimental

Toute réaction nocive et non désirée à un médicament expérimental quelle que soit la dose administrée

- Evènement ou effet indésirable grave

Tout évènement ou effet indésirable qui entraîne la mort, met en danger la vie de la personne qui se prête à la recherche, nécessite une hospitalisation ou la prolongation de l'hospitalisation, provoque une incapacité ou un handicap importants ou durables, ou bien se traduit par une anomalie ou une malformation congénitale, et s'agissant du médicament, quelle que soit la dose administrée.

- Effet indésirable inattendu d'un médicament expérimental

Tout effet indésirable dont la nature, la sévérité ou l'évolution ne concorde pas avec les informations figurant dans le résumé des caractéristiques du produit lorsque le médicament est autorisé, et dans la brochure pour l'investigateur lorsqu'il n'est pas autorisé.

- Fait nouveau

Toute nouvelle donnée de sécurité, pouvant conduire à une réévaluation du rapport des bénéfices et des risques de la recherche ou du médicament expérimental, ou qui pourrait être suffisant pour envisager des modifications dans l'administration du médicament expérimental, dans la conduite de la recherche.

## 8.2 Méthodes et calendrier prévus pour mesurer, recueillir et analyser les paramètres d'évaluation de la sécurité

Comités spécifiques de la recherche

### 8.2.1 Comité de pilotage

Il sera constitué des initiateurs cliniciens du projet, du biostatisticien en charge du projet, des représentants du promoteur nommés pour cette recherche.

Il définira l'organisation générale et le déroulement de la recherche et coordonnera les informations.
Il déterminera initialement la méthodologie et décidera en cours de recherche des conduites à tenir dans les cas imprévus, surveillera le déroulement de la recherche en particulier sur le plan de la tolérance et des évènements indésirables.

### 8.2.2 Suivi des paramètres de sécurité

Dès que les 50 premiers volontaires auront terminé l'étude, l'investigateur principal présentera au chef de projet les données de tolérance du médicament ainsi que le résultat des mesures électrocardiographiques préliminaires. En fonction de cette première analyse, le rythme des compte rendus ultérieurs sera déterminé.

## 8.3 Procédures mises en place en vue de l'enregistrement et de la notification des évènements indésirables

### 8.3.1 Evènements indésirables non graves :

Tout événement indésirable - non grave suivant la définition précédente - observé lors de la recherche et dans ses suites devra être reporté dans le cahier d'observation dans la section prévue à cet effet.

Un seul évènement doit être reporté par item. L'évènement peut correspondre à un symptôme, un diagnostic ou à un résultat d'examen complémentaire jugé significatif. Tous les éléments cliniques ou para-cliniques permettant de décrire au mieux l'évènement correspondant doivent être reportés.

### 8.3.2 Evènements indésirables graves (EIG) : cf annexe 4

Les investigateurs doivent notifier immédiatement au promoteur AP-HP les évènements indésirables graves tels que définis ci-dessus.
L'investigateur complète les formulaires d'évènements indésirables graves (du cahier d'observation de la recherche) et les envoie au DRCD par fax au 01 44 84 17 99 et ce, dans les 48 heures (après si possible un appel téléphonique immédiat au 01 44 84 17 23 en cas de décès ou d'une menace vitale).

Pour chaque évènement indésirable grave, l'investigateur devra émettre un avis sur le lien de causalité de l'évènement avec chaque médicament expérimental et les autres traitements éventuels.

L'obtention d'informations relatives à la description et l'évaluation d'un évènement indésirable peuvent ne pas être possibles dans le temps imparti pour la déclaration initiale.

Aussi, l'évolution clinique ainsi que les résultats des éventuels bilans cliniques et des examens diagnostiques et/ou de laboratoire, ou toute autre information permettant une analyse adéquate du lien de causalité seront rapportés :
soit sur la déclaration initiale d'EIG s'ils sont immédiatement disponibles,
soit ultérieurement et le plus rapidement possible, en envoyant par fax une nouvelle déclaration d'EIG complétée (et en précisant qu'il s'agit d'un suivi d'EIG déclaré et le numéro de suivi).

Toutes les déclarations faites par les investigateurs devront identifier chaque sujet participant à la recherche par un numéro de code unique attribué à chacun d'entre eux.

En cas de décès notifié d'un sujet participant à la recherche, l'investigateur communiquera au promoteur tous les renseignements complémentaires demandés (compte-rendu d'hospitalisation, résultats d'autopsie…).

Tout fait nouveau survenu dans la recherche ou dans le contexte de la recherche, provenant de données de la littérature ou de recherches en cours, devra être notifié au promoteur.

- Déclaration des évènements indésirables graves aux Autorités de Santé
Elle sera assurée par le Pôle de Pharmacovigilance du DRCD, après évaluation de la gravité de l'évènement indésirable, du lien de causalité avec chaque médicament expérimental et les autres traitements éventuels ainsi que du caractère inattendu des effets indésirables.
Toutes les suspicions d'effet indésirable grave inattendu seront déclarées par le promoteur aux autorités compétentes dans les délais légaux.

Toute donnée de sécurité ou tout fait nouveau qui pourrait modifier significativement l'évaluation du rapport des bénéfices et des risques d'un médicament expérimental, ou de la recherche, ou qui pourrait conduire à envisager des modifications concernant l'administration du médicament ou la conduite de la recherche, sera transmise par le promoteur aux autorités compétentes, au Comité de Protection des Personnes et aux investigateurs de la recherche. Par exemple :

a) toute augmentation cliniquement significative de la fréquence d'apparition d'un effet indésirable grave attendu ;
b) des suspicions d'effet indésirable grave inattendu survenus chez des participants ayant terminé l'essai et qui sont notifiés par l'investigateur au promoteur, ainsi que des rapports de suivi éventuels ;

c) tout fait nouveau concernant le déroulement de l'essai clinique ou le développement du médicament, lorsque ce fait nouveau est susceptible de porter atteinte à la sécurité des participants. A titre d'exemple :
un événement indésirable grave susceptible d'être lié aux investigations et aux procédures de diagnostic de l'essai et qui pourrait modifier le déroulement de cet essai,
un risque significatif pour la population de l'essai comme par exemple un manque d'efficacité du médicament utilisé dans le traitement d'une maladie mettant en jeu le pronostic vital,
des résultats significatifs de sécurité issus d'une étude menée chez l'animal récemment terminée (telle qu'une étude de carcinogénicité),
un arrêt anticipé ou une interruption temporaire pour des raisons de sécurité d'un essai conduit avec le même médicament dans un autre pays,
un effet indésirable grave inattendu lié à un médicament non expérimental nécessaire à la réalisation de l'essai (ex : « challenge agents », traitement de secours)
d) tout effet indésirable grave inattendu transmis au promoteur par un autre promoteur d'un essai clinique mené dans un pays tiers portant sur le même médicament.

## 8.4 Modalités et durée du suivi des personnes suite à la survenue d'évènements indésirables

Tout patient présentant un évènement indésirable doit être suivi jusqu'à la résolution ou la stabilisation de celui-ci.

Si l'évènement n'est pas grave, l'évolution en sera notée sur la page correspondante du cahier d'observation à la section prévue à cet effet.

Si l'évènement est grave, un suivi d'EIG sera envoyé au DRCD.

# 9. STATISTIQUES

## 9.1 Description des méthodes statistiques prévues, y compris du calendrier des analyses intermédiaires prévues.

Cette étude porte sur un trait quantitatif, les pools seront réalisés suivant les déciles de la distribution de ce trait après standardisation séparée chez les hommes et chez les femmes. Il est fréquent, comme dans l'étude ayant conduit à l'implication de NOS1AP dans la variabilité de durée de l'intervalle QTc,[15] que les GWAS portant sur des traits quantitatifs utilisent une stratégie en plusieurs étapes commençant par l'étude d'un nombre limité de sujets appartenant aux extrémités de la distribution du trait. Nous proposons d'explorer d'emblée l'ensemble de la distribution sur 500 sujets (10 déciles de 25 sujets pour chaque sexe), ceci aura pour conséquence de générer une variable, codée de 1 à 10 qui permettra d'explorer les queues de distribution (déciles extrêmes) ainsi que des effets plus continus et d'examiner la cohérence des résultats chez les hommes et chez les femmes. Afin de diminuer l'erreur de mesure liée à la constitution des pools, chaque pool sera dupliqué, c'est donc un total de 40 pools de 25 qui seront analysés. Pour chaque pool, les fréquences alléliques (l'analyse par pools ne permet pas d'estimer les fréquences génotypiques) des 317.000 marqueurs de la puce Illumina seront estimées. Les fréquences alléliques des pools correspondant aux extrémités de la distribution du delta-QT standardisé (déciles et quintiles extrêmes successivement) seront comparées par un test de Chi2 à 1 degré de liberté. Dans un deuxième temps, les déciles seront traités comme une variable quantitative 0-10. Il s'agira alors de comparer la médiane de cette variable associée à

chaque allèle. Des techniques de ré-échantillonnage seront utilisées pour générer une distribution de la statistique sous l'hypothèse nulle et tester la significativité des associations. Cette approche sera très puissante pour identifier des facteurs génétiques dont l'effet sur le phénotype est continu. Les analyses seront réalisées chez les hommes et les femmes simultanément. L'homogénéité des associations suivant le sexe sera évaluée dans un deuxième temps.
La puissance d'une étude d'association génétique dépend des fréquences alléliques et des effets phénotypiques associés à ces allèles. Le problème est évidemment compliqué par le nombre considérable de marqueurs explorés dans une GWAS qui expose à un risque d'erreur de 1ère espèce très élévé. La significativité des tests statistiques doit être interprétée en fonction de ce contexte, en tenant compte du « false discovery rate » (FDR). Outre leur significativité statistique, les résultats s'interprètent en fonction de leur cohérence (par exemple effet continu reflété par un changement monotone de fréquence allélique dans les différentes classes phénotypiques) et de leur pertinence biologique. Mais bien évidemment la réplication des résultats dans une étude indépendante est un élément essentiel. C'est en partie dans ce but que 500 sujets supplémentaires seront recrutés. Des études contrôlées ciblées sur des gènes particuliers seront également réalisées. Pour les associations potentiellement les plus intéressantes, un génotypage par analyse individuelle sera réalisé.
L'ensemble des analyses statistiques sera réalisé par un membre de l'équipe de biostatisticiens de l'U525 expert dans l'analyse de GWAS sur ADN poolés.

## 9.2 Nombre prévu de personnes à inclure dans la recherche, et nombre prévu de personnes dans chaque lieu de recherches avec sa justification statistique.

Au cours de la période de 36 mois couvrant le projet, 1000 sujets apparemment sains du point de vue de leur repolarisation ventriculaire seront recrutés et explorés par le CIC de l'hôpital Saint Antoine. Cet effectif est justifié par les contraintes temporelles et budgétaires du présent appel d'offres mais permettra déjà d'identifier des sujets « déviants » dans leur réponse phénotypique. L'objectif global est de constituer à terme une bio-ressource issue de 1000 individus explorés suivant le même protocole.

## 9.3 Degré de signification statistique prévu.

Il sera fixé à $P<0,05$.

## 9.4 Critères statistiques d'arrêt de la recherche.

Cette étude n'évaluant pas l'efficacité du traitement, nous ne prévoyons pas d'analyse intermédiaire.

## 9.5 Méthode de prise en compte des données manquantes, inutilisées ou non valides.

Cette étude se déroulant en quelques heures, nous ne prévoyons pas de procédure de gestion de données manquantes.

## 9.6 Choix des personnes à inclure dans les analyses

Tous les participants seront inclus dans l'analyse.

# 10. DROIT D'ACCES AUX DONNEES ET DOCUMENTS SOURCE

Les personnes ayant un accès direct conformément aux dispositions législatives et réglementaires en vigueur, notamment les articles L.1121-3 et R.5121-13 du code de la santé publique (par exemple, les investigateurs, les personnes chargées du contrôle de qualité, les moniteurs, les assistants de recherche clinique, les auditeurs et toutes personnes appelées à collaborer aux essais) prennent toutes les précautions nécessaires en vue d'assurer la confidentialité des informations relatives aux médicaments expérimentaux, aux essais, aux personnes qui s'y prêtent et notamment en ce qui concerne leur identité ainsi qu'aux résultats obtenus. Les données collectées par ces personnes au cours des contrôles de qualité ou des audits sont alors rendues anonymes.

# 11. CONTROLE ET ASSURANCE DE LA QUALITE

La recherche sera encadrée selon les procédures opératoires standard du promoteur.
Le déroulement de la recherche dans les centres investigateurs et la prise en charge des sujets sera faite conformément à la déclaration d'Helsinki et les Bonnes Pratiques en vigueur.

## 11.1 Procédures de monitoring

Cette étude est classée risque B, correspondant à un risque prévisible proche de celui des soins usuels. La personne chargée du monitoring représentant le promoteur effectuera les tâches suivantes :

- Visite d'ouverture du centre : avant inclusion, pour une mise en place du protocole et prise de connaissance avec les différents intervenants de la recherche biomédicale.

- Lors des visites suivantes, les cahiers d'observation seront revus au fur et à mesure de l'état d'avancement de la recherche par les ARC. L'investigateur principal du centre ainsi que les autres investigateurs qui incluent ou assurent le suivi des personnes participant à la recherche s'engagent à recevoir l' ARC à intervalles réguliers.
Lors de ces visites sur site et en accord avec les Bonnes Pratiques Cliniques, les éléments suivants seront revus :
Respect du protocole et des procédures définies pour la recherche,
Vérification des consentements éclairés des patients
Examen des documents source et confrontation avec les données reportées dans le cahier d'observation quant à l'exactitude, les données manquantes, la cohérence des données selon les règles édictées par les procédures du DRCD.

- Visite de fermeture : récupération des cahiers d'observation, bilan à la pharmacie, documents de la recherche biomédicale, archivage.

## 11.2 Transcription des données dans le cahier d'observation

Toutes les informations requises par le protocole doivent être fournies dans le cahier d'observation et une explication donnée par l'investigateur pour chaque donnée manquante.
Les données devront être transférées dans les cahiers d'observation au fur et à mesure qu'elles sont obtenues qu'il s'agisse de données cliniques ou para-cliniques. Les données devront être copiées de façon nette et lisible à l'encre noire dans ces cahiers (ceci afin de faciliter la duplication et la saisie informatique).

Les données erronées dépistées sur les cahiers d'observation seront clairement barrées et les nouvelles données seront copiées sur le cahier avec les initiales et la date par le membre de l'équipe de l'investigateur qui aura fait la correction.
L'anonymat des sujets sera assuré par un numéro de code et les initiales de la personne qui se prête à la recherche sur tous les documents nécessaires à la recherche, ou par effacement par les moyens appropriés des données nominatives sur les copies des documents source, destinés à la documentation de la recherche.

Les données informatisées sur un fichier seront déclarées à la CNIL selon la procédure adaptée au cas.

# 12. CONSIDERATIONS LEGALES ET ETHIQUES

Le promoteur est défini par la loi 2004-806 du 9 août 2004. Dans cette recherche, l'AP-HP est le promoteur et le Département de la Recherche Clinique et du Développement (DRCD) en assure les missions réglementaires.

## 12.1 Demande d'autorisation auprès de l'Afssaps

Pour pouvoir démarrer la recherche, l'AP-HP en tant que promoteur doit soumettre un dossier de demande d'autorisation auprès de l'autorité compétente l'Afssaps. L'autorité compétente, définie à l'article L. 1123-12, se prononce au regard de la sécurité des personnes qui se prêtent à une recherche biomédicale, en considérant notamment la sécurité et la qualité des produits utilisés au cours de la recherche conformément, le cas échéant, aux référentiels en vigueur, leur condition d'utilisation et la sécurité des personnes au regard des actes pratiqués et des méthodes utilisées ainsi que les modalités prévues pour le suivi des personnes.

## 12.2 Demande d'avis au Comité de Protection des Personnes

En accord avec l'article L.1123-6 du Code de Santé Publique, le protocole de recherche doit être soumis par le promoteur à un Comité de Protection des Personnes L'avis de ce comité est notifié à l'autorité compétente par le promoteur avant le démarrage de la recherche.

## 12.3 Modifications

Le DRCD doit être informé de tout projet de modification du protocole par l'investigateur coordonnateur.
Les modifications devront être qualifiées en substantielles ou non.

Une modification substantielle est une modification susceptible, d'une manière ou d'une autre, de modifier les garanties apportées aux personnes qui se prêtent à la recherche biomédicale (modification d'un critère d'inclusion, prolongation d'une durée d'inclusion, participation de nouveaux centres,…).

Après le commencement de la recherche, toute modification substantielle de celle-ci à l'initiative du promoteur doit obtenir, préalablement à sa mise en oeuvre, un avis favorable du comité et une autorisation de l'autorité compétente. Dans ce cas, si cela est nécessaire, le comité s'assure qu'un nouveau consentement des personnes participant à la recherche est bien recueilli.

Par ailleurs, toute extension de la recherche (modification profonde du schéma thérapeutique ou des populations incluses, prolongation des traitements et ou des actes thérapeutiques non prévus initialement dans le protocole) devra être considérée comme une nouvelle recherche.
Toute modification substantielle devra faire l'objet par le promoteur après paiement d'une taxe d'une demande d'autorisation auprès de l'Afssaps et/ou d'une demande d'avis du CPP.

## 12.4 Déclaration CNIL

La loi prévoit que la déclaration du fichier informatisé des données personnelles collectées pour la recherche doit être faite avant le début effectif de la recherche.

Cette recherche fera l'objet d'une procédure de déclaration simplifiée car les données recueillies dans la recherche sont compatibles avec la liste prévue par la CNIL dans son document de référence.
Le DRCD en qualité de promoteur demandera aux responsables des fichiers informatiques (B.Charbit, J-S Hulot) de s'engager par écrit sur le respect de la méthodologie de référence MR06001 simplifiée.

## 12.5 Note d'information et Consentement éclairé

Il est proposé aux patients et aux témoins d'être inclus dans l'étude après explication et remise d'une information écrite sur le déroulement du protocole. Les participants donneront leur acceptation par écrit.

## 12.6 Rapport final de la recherche

Le rapport final de la recherche sera écrit en collaboration par le coordonnateur et le biostatisticien pour cette recherche. Ce rapport sera soumis à chacun des investigateurs pour avis. Une fois qu'un consensus aura été obtenu, la version finale devra être avalisée par la signature de chacun des investigateurs et adressée au promoteur dans les meilleurs délais après la fin effective de la recherche. Un rapport rédigé selon le plan de référence de l'autorité compétente doit être transmis à l'autorité compétente ainsi qu'au CPP dans un délai de un an, après la fin de la recherche, s'entendant comme la dernière visite de suivi du dernier sujet inclus. Ce délai est rapporté à 90 jours en cas d'arrêt prématuré de la recherche.

# 13. TRAITEMENT DES DONNEES ET CONSERVATION DES DOCUMENTS ET DES DONNEES RELATIVES A LA RECHERCHE

Les documents d'une recherche entrant dans le cadre de la loi sur les recherches biomédicales doivent être archivés par toutes les parties pendant une durée de 15 ans après la fin de la recherche.

Cet archivage indexé comporte :

- Les copies de courrier d'autorisation de l'Afssaps et de l'avis obligatoire du CPP
- Les versions successives du protocole (identifiées par le n° de version et la date de version),
- Les courriers de correspondance avec le promoteur,
- Les consentements signés des sujets sous pli cacheté (dans le cas de sujets mineurs signés par les titulaires de l'autorité parentale) avec la liste ou registre d'inclusion en correspondance,
- Le cahier d'observation complété et validé de chaque sujet inclus,

- Toutes les annexes spécifiques à l'étude,
- Le rapport final de l'étude provenant de l'analyse statistique et du contrôle qualité de l'étude (double transmis au promoteur).
- Les certificats d'audit éventuels réalisés au cours de la recherche

La base de données ayant donné lieu à l'analyse statistique doit aussi faire l'objet d'archivage par le responsable de l'analyse (support papier ou informatique).
La base des données cliniques sera sous la responsabilité du Dr B.Charbit.
La base des données génétiques sera sous la responsabilité du Dr J-S.Hulot.

# 14. ASSURANCE ET ENGAGEMENT SCIENTIFIQUE

## 14.1 Assurance

L'Assistance Publique- Hôpitaux de Paris est le promoteur de cette recherche. En accord avec la loi sur les recherches biomédicales, elle a pris une assurance auprès de la compagnie GERLING KonZern pour toute la durée de la recherche, garantissant sa propre responsabilité civile ainsi que celle de tout intervenant (médecin ou personnel impliqué dans la réalisation de la recherche) (loi n°2004-806, Art L.1121-10 du CSP).
l'Assistance Publique - Hôpitaux de Paris se réserve le droit d'interrompre la recherche à tout moment pour des raisons médicales ou administratives; dans cette éventualité, une notification sera fournie à l'investigateur.

## 14.2 Engagement scientifique

Chaque investigateur s'engagera à respecter les obligations de la loi et à mener la recherche selon les B.P.C., en respectant les termes de la déclaration d'Helsinki en vigueur. Pour ce faire, un exemplaire de l'engagement scientifique (document type DRCD) daté et signé par chaque investigateur de chaque service clinique d'un centre participant sera remis au représentant du promoteur.

# 15. REGLES RELATIVES A LA PUBLICATION

L'AP-HP est propriétaire des données et aucune utilisation ou transmission à un tiers ne peut être effectuée sans son accord préalable.
seront premiers signataires des publications, les personnes ayant réellement participé à l'élaboration du protocole et son déroulement ainsi qu'à la rédaction des résultats. Le Docteur B. Charbit sera premier signataire de la publication finale. Le Docteur J.S. Hulot sera second signataire. Le Docteur F. Cambien sera avant dernier signataire et le Professeur C. Funck-Brentano sera dernier signataire.

L'Assistance Publique- Hôpitaux de Paris doit être mentionnée comme étant le promoteur de la recherche biomédicale et comme soutien financier le cas échéant. les termes « Assistance Publique- Hôpitaux de Paris » doivent apparaître dans l'adresse des auteurs.

# 16. RÉFÉRENCES BIBLIOGRAPHIQUES.


1. Funck-Brentano C. *Pharmacokinetic and pharmacodynamic profiles of d-Sotalol and d,l-Sotalol.* Eur Heart J 1993; 14 Suppl H: 30-35.
2. Demolis JL, Strabach S, Vacheron F, et al. *Assessment of the effect of a single oral dose of telithromycin on Sotalol-induced qt interval prolongation in healthy women.* Br J Clin Pharmacol 2005; 60: 120-127.
3. Funck-Brentano C, Kibleur Y, Le Coz F, et al. *Rate dependence of Sotalol-induced prolongation of ventricular repolarization during exercise in humans.* Circulation 1991; 83: 536-545.
4. Funck-Brentano C, Silberstein DJ, Roden DM, et al. *A mechanism of D-(+)-Sotalol effects on heart rate not related to beta-adrenoceptor antagonism.* Br J Clin Pharmacol 1990; 30: 195-202.
5. Antzelevitch C. *Arrhythmogenic mechanisms of QT prolonging drugs: is QT prolongation really the problem?* J Electrocardiol 2004; 37 Suppl: 15-24.
6. Moss AJ. *Long QT Syndrome.* Jama 2003; 289: 2041-2044.
7. Chiang CE, Roden DM. *The long QT syndromes: genetic basis and clinical implications.* J Am Coll Cardiol 2000; 36: 1-12.
8. Priori SG, Napolitano C, Schwartz PJ. *Low penetrance in the long-QT syndrome: clinical impact.* Circulation 1999; 99: 529-533.
9. Napolitano C, Priori SG, Schwartz PJ, et al. *Genetic testing in the long QT syndrome: development and validation of an efficient approach to genotyping in clinical practice.* Jama 2005; 294: 2975-2980.
10. Shimizu W. *The long QT syndrome: therapeutic implications of a genetic diagnosis.* Cardiovasc Res 2005; 67: 347-356.
11. Priori SG, Schwartz PJ, Napolitano C, et al. *Risk stratification in the long-QT syndrome.* N Engl J Med 2003; 348: 1866-1874.
12. Zareba W, Moss AJ, Schwartz PJ, et al. *Influence of genotype on the clinical course of the long-QT syndrome. International Long-QT Syndrome Registry Research Group.* N Engl J Med 1998; 339: 960-965.
13. Gouas L, Nicaud V, Berthet M, et al. *Association of KCNQ1, KCNE1, KCNH2 and SCN5A polymorphisms with QTc interval length in a healthy population.* Eur J Hum Genet 2005; 13: 1213-1222.
14. Pfeufer A, Jalilzadeh S, Perz S, et al. *Common variants in myocardial ion channel genes modify the QT interval in the general population: results from the KORA study.* Circ Res 2005; 96: 693-701.
15. Arking DE, Pfeufer A, Post W, et al. *A common genetic variant in the NOS1 regulator NOS1AP modulates cardiac repolarization.* Nat Genet 2006; 38: 644-651.
16. Roden DM, Viswanathan PC. *Genetics of acquired long QT syndrome.* J Clin Invest 2005; 115: 2025-2032.
17. Hohnloser SH. *Proarrhythmia with class III antiarrhythmic drugs: types, risks, and management.* Am J Cardiol 1997; 80: 82G-89G.
18. Roden DM. *Drug-induced prolongation of the QT interval.* N Engl J Med 2004; 350: 1013-1022.
19. Shah RR. *Drugs, QTc interval prolongation and final ICH E14 guideline : an important milestone with challenges ahead.* Drug Saf 2005; 28: 1009-1028.
20. Shah RR. *Drug-induced QT interval prolongation: regulatory perspectives and drug development.* Ann Med 2004; 36 Suppl 1: 47-52.
21. Monahan BP, Ferguson CL, Killeavy ES, et al. *Torsades de pointes occurring in association with terfenadine use.* Jama 1990; 264: 2788-2790.

22. Yalta K, Turgut O, Yilmaz A, et al. *Torsades de pointes with a severely prolonged QT interval induced by an initial low dose Sotalol intake.* Int J Cardiol 2007; 116: e95-97.
23. Takenaka K, Ai T, Shimizu W, et al. *Exercise stress test amplifies genotype-phenotype correlation in the LQT1 and LQT2 forms of the long-QT syndrome.* Circulation 2003; 107: 838-844.
24. Shimizu W, Noda T, Takaki H, et al. *Epinephrine unmasks latent mutation carriers with LQT1 form of congenital long-QT syndrome.* J Am Coll Cardiol 2003; 41: 633-642.
25. Kadish AH, Weisman HF, Veltri EP, et al. *Paradoxical effects of exercise on the QT interval in patients with polymorphic ventricular tachycardia receiving type Ia antiarrhythmic agents.* Circulation 1990; 81: 14-19.
26. Haapalahti P, Viitasalo M, Perhonen M, et al. *Ventricular repolarization and heart rate responses during cardiovascular autonomic function testing in LQT1 subtype of long QT syndrome.* Pacing Clin Electrophysiol 2006; 29: 1122-1129.
27. Swan H, Viitasalo M, Piippo K, et al. *Sinus node function and ventricular repolarization during exercise stress test in long QT syndrome patients with KvLQT1 and HERG potassium channel defects.* J Am Coll Cardiol 1999; 34: 823-829.
28. Jouven X, Empana JP, Schwartz PJ, et al. *Heart-rate profile during exercise as a predictor of sudden death.* N Engl J Med 2005; 352: 1951-1958.
29. Le Coz F, Funck-Brentano C, Poirier JM, et al. *Prediction of Sotalol-induced maximum steady-state QTc prolongation from single-dose administration in healthy volunteers.* Clin Pharmacol Ther 1992; 52: 417-426.
30. Malik M. *Problems of heart rate correction in assessment of drug-induced QT interval prolongation.* J Cardiovasc Electrophysiol 2001; 12: 411-420.
31. Wilde AA, Jongbloed RJ, Doevendans PA, et al. *Auditory stimuli as a trigger for arrhythmic events differentiate HERG-related (LQTS2) patients from KVLQT1-related patients (LQTS1).* J Am Coll Cardiol 1999; 33: 327-332.
32. Rautaharju PM, Kooperberg C, Larson JC, et al. *Electrocardiographic predictors of incident congestive heart failure and all-cause mortality in postmenopausal women: the Women's Health Initiative.* Circulation 2006; 113: 481-489.
33. Funck-Brentano C, Jaillon P. *Rate-corrected QT interval: techniques and limitations.* Am J Cardiol 1993; 72: 17B-22B.

# 17. ANNEXES.

## 17.1 Annexe 1 : Carte patient

CARTE PATIENT

*Merci de garder cette carte en permanence avec vous*
*(dans la semaine qui suivra votre participation à l'étude)*

Nom :……………………………………. Prénom : …………………………….

Je participe à la recherche biomédicale : « Identification des sources génétiques de variabilité de l'adaptation de la repolarisation ventriculaire a un stimulus pharmacologique et physiologique dans une population apparemment normale » (GENEREPOL)
dont le promoteur est l'AP-HP

Je reçois le traitement suivant : SOTALOL
80 mg reçu une seule fois le ……./…..../……......

Sous le numéro de patient N° : ………………

Je suis suivi(e) par le Dr……………………………………………………..…….

En cas de nécessité, contacter : l'Hôpital Saint-Antoine (Centre d'investigation clinique)

Dr CHARBIT , Tél. : 01.49.28.24.04 / 06.57.47.94.31 (messagerie)

## 17.2 Annexe 2 : Genome-wide association studies (GWAS)

Traditionally, association studies in complex diseases focused on candidate genes which were *a priori* selected on the basis of their biological function. Thanks to the recent availability of panels of SNPs providing a comprehensive coverage of the genome and to the development of high-throughput genotyping microarrays allowing one to simultaneously test hundreds of thousands, and soon millions of markers, it is now possible to investigate the variability of the genome globally without any prior biological hypothesis. The rationale of this approach is that, if there are unknown disease-causing variants somewhere in the genome, it should be possible to detect them through their LD with genotyped tag SNPs, provided the SNP map is sufficiently dense. This approach is completely independent of traditional biology and offers a great potential for discovering new etiological mechanisms of disease.[1] This is why a great prospect has been put in it, and several large-scale GWA studies of various complex diseases are currently under way.[2] The success of this strategy, however, relies on a number of important conditions : 1. the existence of susceptibility genes having a detectable marginal effect since GWA studies are not well adapted to the detection of complex interactions; 2. the underlying postulate that the genotyped tag SNPs are able to capture the causative variant by LD, which may be an issue for variants with a low frequency or located in a region of weak LD; 3. the ability to replicate the findings in other studies to minimize the false positives which will inevitably be generated; 4. a rigorous characterization of phenotypes in order to minimize the clinical heterogeneity of the disease.

The GWA approach has already demonstrated its power for identifying genes contributing to complex diseases such as macular degeneration,[3] memory performance, [4] type II diabetes,[5,6] inflammatory bowel disease[7] and electrocardiographic QT interval. [8] But as for candidate gene association studies, it is likely that the multiplication of GWA studies will generate results that will not be replicated [9].

*NOS1AP*. Long QT syndrome (LQTS) is a rare monogenic disease responsible for sudden cardiac death caused by mutations in ion channel genes. There are seven LQTS-related genes identified so far (LQT1 to LQT7) that bear at least 300 different mutations, the two most commonly involved genes being LQT1 (*KCNQ1*) and LQT2 (*KCNH2*).[10] Unravelling the genetic basis of the LQTS has contributed in an important way to the understanding of the fundamental mechanisms underlying human cardiac electrophysiology.[10] However, the interest for the QT interval (QTi) and its genetics goes beyond the rare LQTS. A QTi prolongation diagnosed on the ECG is an indicator of delayed ventricular repolarization which may become clinically manifest with the occurrence of syncopes and ventricular arrhythmias such as torsades de pointe that may lead to sudden death. The QTi length has an heritability of 30%, and short as well as long QTis are associated with an increased risk of cardiovascular morbidity and mortality. Recently, a GWA approach was used to identify genes whose variability may contribute to the QTi variability in the general population.[8] A multistage design was used, starting with the comparison of the two extremes of the QTi distribution in females, followed by a step of refining the best regions of interest, and finally replicating the most interesting result in the whole initial population and two independent studies. This strategy resulted in the identification of frequent polymorphisms in the nitric oxide synthase 1 adaptor protein (*NOS1AP*) gene that are consistently associated with QTi both in men and women. NOS1AP is a regulator of the neuronal nitric oxide synthase that had not been previously suspected to be involved in cardiac repolarization. Its genetic variability accounts for approximately 1.5% of the variance of the QTi in the population at large. The finding which has now been replicated in several large scale studies (yet unpublished) demonstrates the potential of the GWA approach for the discovery of new disease pathways and opens a new area of research for the prevention of sudden death.

- DNA pooling and GWA

A major limitation of the GWA approach is that it is very cost- and time-consuming when applied to studies of large sample size. One solution to circumvent this limitation is to pool DNA samples.[11] This is possible because the general principle of SNP microarray technology is to produce a quantitative signal proportional to the number of copies of a given allele in the DNA sample analyzed. When the DNA sample is that of an individual subject, the signal is used to classify subjects into three genotypic categories according to their number of copies (0, 1 or 2) of a given SNP allele. When samples correspond to pooled DNAs, the signal indicates the number of copies of a given allele within the group of subjects that compose the pool. By judiciously composing the pools – for example grouping cases and controls in two distinct pools - it is possible to compare allele frequencies between pools. This economical approach to the estimation of allele frequencies in GWA studies has proven to be efficient.[4,12] The consequence of this is that the GWA approach should not be reserved to highly funded programs. All studies in which DNA is available could benefit from this approach, the major restriction being the relevance of the study and the ability to construct pools of high quality. The possibility to combine the results of numerous studies should compensate for the mild loss of precision incurred by the pooling approach while facilitating replication analysis.

- References

1. Hirschhorn JN, Daly MJ. Genome-wide association studies for common diseases and complex traits. *Nat Rev Genet*. 2005;6:95-108.

2. Thomas DC, Haile RW, Duggan D. Recent developments in genomewide association scans: a workshop summary and review. *Am J Hum Genet*. 2005;77:337-45.

3. Klein RJ, Zeiss C, Chew EY, Tsai JY, Sackler RS, Haynes C, Henning AK, SanGiovanni JP, Mane SM, Mayne ST, Bracken MB, Ferris FL, Ott J, Barnstable C, Hoh J. Complement factor H polymorphism in age-related macular degeneration. *Science*. 2005;308:385-9.

4. Papassotiropoulos A, Stephan DA, Huentelman MJ, Hoerndli FJ, Craig DW, Pearson JV, Huynh KD, Brunner F, Corneveaux J, Osborne D, Wollmer MA, Aerni A, Coluccia D, Hanggi J, Mondadori CR, Buchmann A, Reiman EM, Caselli RJ, Henke K, de Quervain DJ. Common Kibra alleles are associated with human memory performance. *Science*. 2006;314:475-8.
5. Grant SFA, Thorleifsson G, Reynisdottir I, Benediktsson R, Manolescu A, Sainz J, Helgason A, Stefansson H, Emilsson V, Helgadottir A, Styrkarsdottir U, Magnusson KP, Walters GB, Palsdottir E, Jonsdottir T, Gudmundsdottir T, Gylfason A, Saemundsdottir J, Wilensky RL, Reilly MP, Rader DJ, Bagger Y, Christiansen C, Gudnason V, Sigurdsson G, Thorsteinsdottir U, Gulcher JR, Kong A, Stefansson K. Variant of transcription factor 7-like 2 (TCF7L2) gene confers risk of type 2 diabetes. *Nat Genet*. 2006;38:320-3.
6. Sladek, R., Rocheleau, G., Rung, J., Dina, C., Shen, L., Serre, D., et coll. (2007). A genome-wide association study identifies novel risk loci for type 2 diabetes. *Nature*, *445*(7130), 881-5
7. Duerr RH, Taylor KD, Brant SR, Rioux JD, Silverberg MS, Daly MJ, Steinhart AH, Abraham C, Regueiro M, Griffiths A, Dassopoulos T, Bitton A, Yang H, Targan S, Datta LW, Kistner EO, Schumm LP, Lee AT, Gregersen PK, Barmada MM, Rotter JI, Nicolae DL, Cho JH. A genome-wide association study identifies IL23R as an inflammatory bowel disease gene. *Science*. 2006;314:1461-3.
8. Arking DE, Pfeufer A, Post W, Kao WHL, Newton-Cheh C, Ikeda M, West K, Kashuk C, Akyol M, Perz S, Jalilzadeh S, Illig T, Gieger C, Guo C-Y, Larson MG, Wichmann HE, Marban E, O'Donnell CJ, Hirschhorn JN, Kaab S, Spooner PM, Meitinger T, Chakravarti A. A common genetic variant in the NOS1 regulator NOS1AP modulates cardiac repolarization. *Nat Genet*. 2006;38:644-51.
9. Herbert, A., Gerry, N.P., McQueen, M.B., Heid, I.M., Pfeufer, A., Illig, T., et coll. (2006). A common genetic variant is associated with adult and childhood obesity. *Science (New York, N.Y.)*, *312*(5771), 279-83. (See also comments Science. 2007 Jan 12;315(5809):187)
10. Moss AJ, Kass RS. Long QT syndrome: from channels to cardiac arrhythmias. *J Clin Invest*. 2005;115:2018-24.
11. Sham P, Bader JS, Craig I, O'Donovan M, Owen M. DNA Pooling: a tool for large-scale association studies. *Nat Rev Genet*. 2002;3:862-71.
12. Meaburn E, Butcher LM, Schalkwyk LC, Plomin R. Genotyping pooled DNA using 100K SNP microarrays: a step towards genomewide association scans. *Nucleic Acids Res*. 2006;34:e27.

## 17.3 Annexe 3 : Annonce de recrutement

### Annonce de recrutement pour la presse

Le Centre d'Investigation Clinique de l'Hôpital Saint-Antoine recherche des hommes et des femmes pour participer à une étude indemnisée d'une journée.
Contacter le 01.49.28.24.04

### Annonce de recrutement pour affichage local

Le Centre d'Investigation Clinique de l'Hôpital Saint-Antoine recherche des hommes et des femmes pour participer à une étude indemnisée d'une journée.
Contacter le 01.49.28.24.04

## 17.4 Annexe 4 : Formulaire de déclaration d'EIG

| |
|---|
| FICHE D'OBSERVATION D'UN EVENEMENT INDESIRABLE GRAVE SUSCEPTIBLE D'ETRE DU **A UNE RECHERCHE BIOMEDICALE SUR UN MEDICAMENT OU PRODUIT ASSIMILE** |

ASSISTANCE PUBLIQUE HÔPITAUX DE PARIS

Cette fiche doit être retournée dûment complétée recto-verso à la DRRC
par fax : 01 44 84 17 99

date de déclaration : |__|__| |__|__| |__|__|__|__|
jj mm aaaa

Titre de l'essai clinique : **IDENTIFICATION DES SOURCES GENETIQUES DE VARIABILITE DE L'ADAPTATION DE LA REPOLARISATION VENTRICULAIRE A UN STIMULUS PHARMACOLOGIQUE ET PHYSIOLOGIQUE DANS UNE POPULATION APPAREMMENT NORMALE**

Nom et adresse du centre : CIC Saint-Antoine 184 rue du faubourg Saint-Antoine 75012 Paris 01.49.28.24.04

Investigateur : ______________________________

**Identification du patient :**

Nom : |__|__|__|__| Prénom : |__|__|

Patient n° : |__|__|__|__|__|__|

Date de naissance : |__|__| |__|__| |__|__|__|__|

Age : |__|__| ans

Poids : |__|__|__| kg

Taille : |__|__|__| cm

Sexe : ☐ Masculin ☐ Féminin

***Evénement indésirable grave :***

- Décès ☐
- mise en jeu du pronostic vital ☐
- Nécessite ou prolonge l'hospitalisation ☐
- Séquelles durables ☐
- Anomalie congénitale ☐
- autre (préciser)______________________ ☐

______________________________

Antécédents (allergie, insuffisance rénale …) : ______________________________

______________________________

______________________________

Description de l'événement indésirable :

Date de survenue : |__|__| |__|__| |__|__|__|__| et heure de survenue : |__|__| |__|__|
jj mm aaaa hh min

______________________________

______________________________

______________________________

Délai de survenue après la dernière prise : ______________________________

Médicaments de l'essai administrés avant la survenue de l'événement indésirable :

| nom commercial ou DCI | voie | dose/24h | date début | date fin (si arrêt) sinon en cours |
|---|---|---|---|---|
| | | | | |
| | | | | |
| | | | | |

Médicaments associés (à l'exclusion de ceux utilisés pour traiter l'événement) :

| nom commercial ou DCI | voie | dose/24h | date début | date fin (si arrêt) sinon en cours | indication |
|---|---|---|---|---|---|
| | | | | | |
| | | | | | |
| | | | | | |
| | | | | | |

| | | | | | |
|---|---|---|---|---|---|
| | | | | | |

Evolution *(indiquez si des mesures symptomatiques ont été prises) :*

______________________________________________

______________________________________________

______________________________________________

______________________________________________

Date de disparition : |__|__| |__|__| |__|__|__|__| et heure de disparition : |__|__| |__|__|

jj mm aaaa hh min

Autres étiologies envisagées, examens complémentaires réalisés et résultats :

______________________________________________

______________________________________________

______________________________________________

Traitements de l'essai :

levée de l'aveugle : ☐ non ☐ oui ☐ sans objet date : |__|__| |__|__| |__|__|__|__|

résultat de la levée de l'aveugle : ______________________

réadministration du (des) médicament(s) : ☐ non ☐ oui ☐ sans objet date : |__|__| |__|__| |__|__|__|__|

si oui, lesquels : ______________________________

______________________________________________

récidive après réadministration : ☐ non ☐ oui ☐ sans objet date : |__|__| |__|__| |__|__|__|__|

Selon l'investigateur, l'événement indésirable semble plutôt lié :

☐ au(x) médicaments de l'essai ☐ aux procédures de l'essai

☐ au(x) médicaments associés ☐ à la progression de la maladie

☐ à une maladie intercurrente ☐ autre, préciser : ______________

Date : |__|__| |__|__| |__|__|__|__| Nom : Signature :

PARTIE RESERVEE AU PROMOTEUR : NE PAS REMPLIR

*Numéro d'identification de l'événement :* n°

date de réception par le promoteur : |__|__| |__|__| |__|__|__|__|

date de ce rapport : |__|__| |__|__| |__|__|__|__| ☐ initial ☐ suivi n°

Selon le promoteur, l'événement indésirable semble plutôt lié :

☐ au(x) médicament(s) étudié(s) ☐ aux procédures de l'essai

☐ à une médication concomitante ☐ à la progression de la maladie

☐ à une maladie intercurrente ☐ autre, préciser : ______________

Si selon le promoteur, l'événement semble plutôt lié au médicament :

☐ l'événement indésirable est attendu ☐ l'événement indésirable est inattendu

Commentaires du promoteur :

______________________________________________

______________________________________________

Nom et qualité du représentant du promoteur : Le représentant légal de la DRRC

Signature :

## 17.5 Annexe 5 : Annexe pharmaceutique et fiche Vidal du Sotalol

**Annexe pharmaceutique**
**Contacts pharmaceutiques et circuit du médicament**

**Essai GENEREPOL**
**Promoteur : AP-HP**
**Investigateur coordonnateur : Dr Beny CHARBIT**

**Identification des sources génétiques de variabilité de l'adaptation de la repolarisation ventriculaire à un stimulus pharmacologique et physiologique dans une population apparemment normale.**

**Contacts**
Pharmacie Hôpital Saint Antoine :
Anne Daguenel-Nguyen : 01 49 28 28 47 Fax 2142
Internes essais cliniques : 01 49 28 32 74
Secrétariat : Muriel Dabout : 01 49 28 30 34

**Produits de l'essai**
Présentation :
Sotalol 80 mg comprimé sécable
Substance active : sotalol chlorhydrate.
boite de 30cpr

Posologie et schéma thérapeutique :
Sotalol 80mg : 1cpr par patient pour l'étude (monoprise)

**Documents et Matériel**

1/DOSSIER DE LOT : constitué :
d'une fiche de reconditionnement par lot de 60 patients
d'une fiche de reconditionnement pour la réalisation des lots de secours
2/Etiquettes
3/Ordonnancier de fabrication des lots pour essai clin.

**Présentation des lots**

Des enveloppes contenant un comprimé de sotalol 80mg pour une prise destinée à un patient seront préparées à l'avance par la pharmacie.
Les campagnes de reconditionnement se feront par 60 comprimés.
Nombre de sujets : 1000 patients
Enveloppe de secours :
A chaque reconditionnement de 2 boites de 30cpr de Sotalol :
5 enveloppes de secours seront préparées systématiquement.

**Modalités**
-Commande des produits à l'AGEPS au tri et facturation au service **UA 3021**
-Préparation des séries d'enveloppes par 55 + 5 de secours à la demande du CIC par la pharmacie.
-Constitution d' une fiche de reconditionnement par campagne de 55 patients et d'une spécifique pour les lots de secours.
-Etiquetages des enveloppes (nom de l'étude , lieu de la réalisation des lots, n° patient, N° lot, date de péremption) + mentions Art R5123 CSP)
les enveloppes de secours seront identifiables.

| Rédacteur :<br>Anne Daguenel-Nguyen | lecteur<br>Pour la Pharmacie | Lecteur<br>pour CIC | Approbateur : Dr Charbit |
|---|---|---|---|
| Date :<br>Visa : | | | |

-Libération des lots par la pharmacie
-Inscription à l'ordonnancier

**Dispensation,**

Globale au service des 55 enveloppes + 5 enveloppes de secours contre une ordonnance libre mentionnant le nom de l'étude.

**Suivi de l'administration - gestion des retours à la pharmacie du centre investigateur**.

Par le CIC.
**Devenir des médicaments en fin d'essai**

Les médicaments retournés à la pharmacie sont conservés jusqu'à rédaction du rapport final de l'étude. Ils seront détruits à la pharmacie (certificat de destruction).

Etiquette Enveloppe :

Etude GENEREPOL Dr Charbit CIC
Service Pharmacie Hôpital Saint Antoine Paris XII
Tel : 01 49 28 28 47
**Sotalol 80mg 1cpr** N°patient : /____________/
**N° de patient : ________** Date administration :____________
N° de lot : ________Date de péremption :___________
Conservation à température ambiante
*« utilisation sous stricte surveillance médicale (Art R 5123 CSP)*
*Médicament sous essais clinique.*

Etude GENEREPOL Dr Charbit CIC
Service Pharmacie Hôpital Saint Antoine Paris XII
Tel : 01 49 28 28 47

**Enveloppe de Secours:** Sotalol 80 mg 1cpr N°patient : /______________/

N° de lot : ________Date de péremption :___________
Conservation à température ambiante
*« utilisation sous stricte surveillance médicale (Art R 5123 CSP)*
*Médicament sous essais clinique.*

| Rédacteur :<br>Anne Daguenel-Nguyen | lecteur<br>Pour la Pharmacie | Lecteur<br>pour CIC | Approbateur : Dr Charbit |
|---|---|---|---|
| Date :<br>Visa : | | | |

# Fiche Vidal du Sotalol

## SOTALOL EG 80 mg cp séc

**FORME** comprimé sécable

**COMPOSITION**

| | par cp |
|---|---|
| **sotalol chlorhydrate** | 80 mg |

*Excipients : lactose monohydrate, amidon de maïs, cellulose microcristalline, magnésium stéarate, acide stéarique, silice colloïdale anhydre.*

**INDICATIONS**

Prévention des récidives des :
- tachycardies ventriculaires menaçant le pronostic vital : le traitement doit être instauré en milieu hospitalier sous monitorage,
- tachycardies ventriculaires documentées symptomatiques et invalidantes en l'absence d'insuffisance cardiaque non contrôlée,
- tachycardies supraventriculaires documentées en l'absence d'insuffisance cardiaque non contrôlée lorsque la nécessité d'un traitement est établie.

**POSOLOGIE et MODE D'ADMINISTRATION**

L'initiation du traitement ou le changement de posologie se feront après une évaluation médicale appropriée incluant contrôle ECG avec mesure du QT corrigé ainsi que le dosage de la kaliémie, évaluation de la fonction rénale et en tenant compte des traitements associés.
Comme avec les autres agents antiarythmiques, il est recommandé que Sotalol EG soit initié et les doses augmentées sous surveillance ECG, car les effets proarythmiques peuvent survenir non seulement à l'initiation du traitement mais également à chaque changement de posologie.
La dose initiale sera de 80 mg en 1 ou 2 prises. La posologie doit être adaptée avec un intervalle de 2 ou 3 jours entre chaque augmentation afin d'obtenir l'état d'équilibre et permetttre la surveillance de l'intervalle QT.
La plupart des patients répondent à une posologie, comprise entre 160 et 320 mg en 2 prises par jour.
Chez certains patients ayant des troubles menaçant le pronostic vital, la posologie pourra être portée à 480 mg voire 640 mg. Pour ces patients, le rapport bénéfice/risque sera évalué.
- Chez l'insuffisant rénal, la posologie sera ajustée en fonction de la clairance de la créatinine, le sotalol étant excrété principalement par le rein.

| Clairance de la créatinine (ml/min) | Posologie recommandée |
|---|---|
| > 60 | dose usuelle |
| 30-60 | demi-dose |
| 10-30 | quart de dose |
| < 10 | éviter le traitement |

Formule de Cockcroft et Gault :
Homme : ((140 - âge) × poids (kg))/(72 × créatinine (mg/dl))
Femme : idem × 0,85.
Si la créatinine est exprimée en µmol/l, diviser sa valeur par 88,4 (1 mg/dl = 88,4 µmol/l).
- Chez l'insuffisant hépatique, aucun ajustement posologique n'est nécessaire.

**CONTRE-INDICATIONS**

**Absolue(s) :**

Allongement de l' Espace QT, Torsades de pointes, Hypersensibilité aux bêtabloquants, Hypersensibilité à l'un des autres composants, Asthme, Bronchopneumopathie chronique obstructive, Insuffisance cardiaque décompensée, Choc cardiogénique, Bloc auriculoventriculaire de 2e degré non appareillé, Bloc auriculoventriculaire de 3e degré non appareillé, Angor spastique, Sinus auriculaire non appareillé, maladie (du), Bradycardie, Raynaud, syndrome (de), Circulation périphérique, trouble sévère (de la), Phéochromocytome non traité, Hypotension artérielle, Anesthésie entraînant une dépression myocardique, Insuffisance rénale très sévère : clairance de la créatinine < 10 ml/ min, Acidose métabolique, Intolérance génétique au galactose, Lactase,déficit (en), Malabsorption du glucose et du galactose, syndrome (de)

**Relative(s) :**

- Allaitement

**MISES EN GARDE et PRECAUTIONS D'EMPLOI**

- Traitement à arrêter progressivement
  Ne jamais interrompre brutalement le traitement chez les angineux : l'arrêt brusque peut entraîner des troubles du rythme graves, l'infarctus du myocarde et la mort subite.

- Angor
  Ne jamais interrompre brutalement le traitement chez les angineux : l'arrêt brusque peut entraîner des troubles du rythme graves, l'infarctus du myocarde et la mort subite.

- Risque d'arythmie
  L'effet indésirable le plus sérieux des médicaments antiarythmiques est l'aggravation des arythmies pré-existantes ou le déclenchement de nouveaux troubles du rythme.
  Les produits qui allongent l'intervalle QT, dont le sotalol peuvent provoquer des torsades de pointes.
  Les facteurs favorisant cet effet ont été identifiés :
  . intervalle QT spontanément long (> 450 ms) avant le traitement,
  . bradycardies (< 60 battements par minute),
  . hypokaliémie ou hypomagnésémie, (notamment sous diurétiques proximaux),
  . taux sériques élevés de sotalol, soit par surdosage, soit par accumulation au cours d'une insuffisance rénale,
  . associations à d'autres produits favorisant des torsades de pointes (cf Contre-indication et Interactions),
  . troubles du rythme ventriculaires graves,
  . les femmes paraissent avoir un risque accru de survenue de torsades de pointes.
  Les torsades de pointes sont dose-dépendantes et de ce fait surviennent le plus souvent lors de l'initiation du traitement ou lors d'une augmentation de la posologie. Elles peuvent être symptomatiques (syncopes), elles peuvent régresser spontanément, plus rarement dégénérer en fibrillation ventriculaire.
  Des essais cliniques chez des patients ayant des troubles du rythme ventriculaire comportant un risque vital (tachycardies ventriculaires soutenues ou fibrillations ventriculaires), l'incidence des pro-arythmies sévères (torsades de pointes ou nouvelles tachycardies ventriculaires ou fibrillations ventriculaires) fut inférieure à 2 % à des doses <= à 320 mg. L'incidence a plus que doublé pour des posologies plus élevées.
  Les patients qui présentent le risque le plus marqué de développer des effets pro-arythmiques graves sous Sotalol (7 %) sont ceux qui ont une tachycardie ventriculaire soutenue et une insuffisance cardiaque. Une initiation du traitement à 80 mg et une adaptation posologique progressive permettent de réduire le risque de survenue d'effets pro-arythmiques.
  Un allongement de QTc supérieur à 480 ms nécessite une surveillance étroite et une réévaluation du rapport bénéfice/risque.

- Risque d'aggravation de l'arythmie
  L'effet indésirable le plus sérieux des médicaments antiarythmiques est l'aggravation des arythmies pré-existantes ou le déclenchement de nouveaux troubles du rythme.
  Les produits qui allongent l'intervalle QT, dont le sotalol peuvent provoquer des torsades de pointes.
  Les facteurs favorisant cet effet ont été identifiés :
  . intervalle QT spontanément long (> 450 ms) avant le traitement,
  . bradycardies (< 60 battements par minute),
  . hypokaliémie ou hypomagnésémie, (notamment sous diurétiques proximaux),
  . taux sériques élevés de sotalol, soit par surdosage, soit par accumulation au cours d'une insuffisance rénale,
  . associations à d'autres produits favorisant des torsades de pointes (cf Contre-indication et Interactions),
  . troubles du rythme ventriculaires graves,
  . les femmes paraissent avoir un risque accru de survenue de torsades de pointes.
  Les torsades de pointes sont dose-dépendantes et de ce fait surviennent le plus souvent lors de l'initiation du traitement ou lors d'une augmentation de la posologie. Elles peuvent être symptomatiques (syncopes), elles peuvent régresser spontanément, plus rarement dégénérer en fibrillation ventriculaire.
  Des essais cliniques chez des patients ayant des troubles du rythme ventriculaire comportant un risque vital (tachycardies ventriculaires soutenues ou fibrillations ventriculaires), l'incidence des pro-arythmies sévères (torsades de pointes ou nouvelles tachycardies ventriculaires ou fibrillations ventriculaires) fut inférieure à 2 % à des doses <= à 320 mg. L'incidence a plus que doublé pour des posologies plus élevées.
  Les patients qui présentent le risque le plus marqué de développer des effets pro-arythmiques graves sous Sotalol (7 %) sont ceux qui ont une tachycardie ventriculaire soutenue et une insuffisance cardiaque. Une initiation du traitement à 80 mg et une adaptation posologique progressive permettent de réduire le risque de survenue d'effets pro-arythmiques.
  Un allongement de QTc supérieur à 480 ms nécessite une surveillance étroite et une réévaluation du rapport bénéfice/risque.

- Risque de bradycardie
  Si la fréquence s'abaisse au-dessous de 50 à 55 pulsations par minute au repos et que le patient présente des symptômes liés à la bardycardie, la posologie doit être diminuée. La bradycardie majore le risque de survenue de torsades de pointes.

- Bloc auriculoventriculaire du premier degré
  Etant donné son effet dromotrope négatif, le sotalol doit être administré avec prudence aux patients présentant un bloc auriculo-ventriculaire du premier degré.

- Insuffisance cardiaque
  Des précautions lors de l'initiation et l'ajustement posologique avec le sotalol chez les patients avec dysfonction ventriculaire gauche contrôlé par un traitement (tel que inhibiteur de l'enzyme de conversion, diurétique, digitalique,...) doivent être prises. Le sotalol du fait de ces propriétés bêta-bloquantes peut diminuer la contractilité myocardique et entraîner une décompensation brutale de l'insuffisance cardiaque sévère.

- Infarctus du myocarde, antécédent récent
  Chez les patients en post-infarctus, avec dysfonction ventriculaire gauche, le bénéfice/risque d'un traitement par le sotalol doit être évalué. Si ce traitement s'avère nécessaire, l'initiation ainsi que l'adaptation posologique doivent être étroitement suivies. Le sotalol devra être évité chez les patients ayant une fraction d'éjection <= 40 % sans aryhmie ventriculaire grave.

- Hypokaliémie
  Le sotalol ne devra pas être utilisé chez les patients présentant une hypokaliémie ou une hypomagnésémie avant correction préalable du déséquilibre. L'équilibre électrolytique et acido-basique sera particulièrement suivi chez les patients en cas de diarrhée sévère ou prolongée ou chez les sujets recevant un traitement hypokaliémiant ou hypomagnésémiant.

- Hypomagnésémie
  Le sotalol ne devra pas être utilisé chez les patients présentant une hypokaliémie ou une hypomagnésémie avant correction préalable du déséquilibre. L'équilibre électrolytique et acido-basique sera particulièrement suivi chez les patients en cas de diarrhée sévère ou prolongée ou chez les sujets recevant un traitement hypokaliémiant ou hypomagnésémiant.

- Diarrhée
  Le sotalol ne devra pas être utilisé chez les patients présentant une hypokaliémie ou une hypomagnésémie avant correction préalable du déséquilibre. L'équilibre électrolytique et acido-basique sera particulièrement suivi chez les patients en cas de diarrhée sévère ou prolongée ou chez les sujets recevant un traitement hypokaliémiant ou hypomagnésémiant.

- Sujet allergique
  Chez les patients susceptibles de faire une réaction anaphylactique sévère, qu'elle qu'en soit l'origine, en particulier avec des produits de contraste iodés ou la floctafénine (cf Interactions) ou en cours de traitement désensibilisant, le sotalol, en raison de ses propriétés bêta-bloquantes, peut entraîner une aggravation de la réaction et une résistance à son traitement par l'adrénaline aux posologies habituelles.
- Traitement de désensibilisation en cours
  Chez les patients susceptibles de faire une réaction anaphylactique sévère, qu'elle qu'en soit l'origine, en particulier avec des produits de contraste iodés ou la floctafénine (cf Interactions) ou en cours de traitement désensibilisant, le sotalol, en raison de ses propriétés bêta-bloquantes, peut entraîner une aggravation de la réaction et une résistance à son traitement par l'adrénaline aux posologies habituelles.
- Thyrotoxicose
  Le sotalol, en raison de ses propriétés bêta-bloquantes, est susceptible d'en masquer les signes cardiovasculaires.
- Psoriasis
  Des aggravations de la maladie ayant été rapportées sous bêta-bloquant, l'indication mérite d'être pesée.
- Sujet âgé
  Le respect absolu des contre-indications classiques est impératif. On veillera à initier le traitement par une posologie faible et à assurer une surveillance étroite.
- Insuffisance rénale
  En cas d'insuffisance rénale, il convient d'adapter la posologie à l'état de la fonction rénale.
- Diabète
  Prévenir le malade et renforcer en début de traitement l'autosurveillance glycémique. Les signes annonciateurs d'une hypoglycémie peuvent être masqués, en particulier tachycardie, palpitations et sueurs.
- Risque d'allongement de l'espace QT
  Un allongement QTc (> 480 ms) nécessite une surveillance étroite et une réévaluation du rapport bénéfice/risque. Le risque de torsades de pointes est proportionnel au degré d'allongement du QT.
- Anesthésie générale
  Du fait de ses propriétés bêta-bloquantes, le sotalol peut entraîner une atténuation de la tachycardie réflexe et une augmentation du risque d'hypotension. La poursuite du traitement par le sotalol diminue le risque d'arythmie, d'ischémie myocardique et de poussées hypertensives. Il convient de prévenir l'anesthésiste que le patient est traité par le sotalol.
  Si l'arrêt du traitement est jugé nécessaire, une suspension de 48 heures est considérée comme suffisante pour permettre la réapparition de la sensibilité aux catécholamines.
  Dans certains cas, le traitement par le sotalol ne peut être interrompu.
  Chez les malades atteints d'insuffisance coronaire, il est souhaitable de poursuivre le traitement jusqu'à l'intervention, étant donné le risque lié à l'arrêt brutal des bêta-bloquants.
  En cas d'urgence ou d'impossibilité d'arrêt, le patient doit être protégé d'une prédominance vagale par une prémédication suffisante d'atropine renouvelée selon les besoins.
  L'anesthésie devra faire appel à des produits aussi peu dépresseurs myocardiques que possibles et les pertes sanguines devront être compensées.
- Sportif
  L'attention des sportifs sera attirée sur le fait que cette spécialité contient un principe actif pouvant induire une réaction positive des tests pratiqués lors des contrôles antidopages.

**GROSSESSE ET ALLAITEMENT**

**Grossesse :**

Les études chez l'animal n'ont pas mis en évidence d'effet tératogène. En l'absence d'effet tératogène chez l'animal, un effet malformatif dans l'espèce humaine n'est pas attendu. En effet, à ce jour, les substances responsables de malformations dans l'espèce humaine se sont révélées tératogènes chez l'animal au cours d'études bien conduites sur deux espèces.
En clinique, aucun effet tératogène n'a été rapporté à ce jour et les résultats d'études prospectives contrôlées avec quelques bêta-bloquants n'ont pas fait état de malformations à la naissance.
Chez le nouveau-né de mère traitée, l'action bêta-bloquante persiste plusieurs jours après la naissance et peut se traduire par une bradycardie, une détresse respiratoire, ou une hypoglycémie ; mais le plus souvent, cette rémanence est sans conséquence clinique.
Il peut néanmoins survenir, par réduction des réactions cardiovasculaires de compensation, une défaillance cardiaque nécessitant une hospitalisation en soins intensifs (cf Surdosage), tout en évitant les solutés de remplissage (risque d'OAP).
En conséquence, ce médicament, dans les conditions normales d'utilisation, peut être prescrit pendant la grossesse si besoin. En cas de traitement jusqu'à l'accouchement, une surveillance attentive du nouveau-né (fréquence cardiaque et glycémie pendant les 3 à 5 premiers jours de vie) est recommandée.

**Allaitement :**

Les bêta-bloquants sont excrétés dans le lait (cf Pharmacocinétique).
La survenue d'hypoglycémie et de bradycardie a été décrite pour certains bêta-bloquants peu liés aux protéines plasmatiques. En conséquence, l'allaitement est déconseillé en cas de nécessité de traitement.

**EFFETS INDESIRABLES**

Arythmie(aggravation), Torsades de pointes, Bradycardie, Dyspnée, Douleur thoracique, Palpitation, Œdème, Electrocardiogramme(anomalie), Hypotension artérielle, Arythmie, Syncope, Insuffisance cardiaque, Syndrome de Raynaud, Claudication intermittente(aggravation), Eruption psoriasiforme, Psoriasis(aggravation), Nausée, Vomissement, Diarrhée, Dyspepsie, Douleur abdominale, Flatulence, Crampe, Asthénie, Vertige, Céphalée, Dépression, Insomnie, Paresthésie, Anxiété, Anticorps antinucléaire(augmentation) (Rare), Syndrome lupique (Exceptionnel)

**PHARMACODYNAMIE**

**Classement ATC :**
C07AA07 / SOTALOL
**Classement Vidal :**
Antiarythmique groupe II : bêtabloquant (sotalol)

**PRESENTATIONS**

**Liste I**

**CIP : 3549057** (SOTALOL EG 80 mg cp séc : B/30).
Disponibilité : officines
Remboursé à 65 %, Agréé aux collectivités

## 17.6 Annexe 6 : Prélèvements sanguins

- La kaliémie sera dosée en routine par le laboratoire de Biochimie de l'hôpital Saint-Antoine

- Le dosage du Sotalol sera prélevé sur un tube héparinate de Lithium de 5 ml, centrifugé (3500 tours/minute pendant 10 minutes à température ambiante), aliquoté puis congelé à –20 °C.

  Les échantillons plasmatiques seront groupés et adressés au laboratoire de dosage de l'hôpital Raymond Poincaré en fin d'étude par envoi réfrigéré puis détruits après dosage.

- Le prélèvement de génotypage pour extraction d'ADN sera de 20 ml et acheminé au laboratoire de dosage de l'hôpital Pitié Salpêtrière par le tri-labo.
  L'ADN sera conservé jusqu'à épuisement de l'échantillon.
